# Quantifying the Contribution of the Liver to Glucose Homeostasis: A Detailed Kinetic Model of Human Hepatic Glucose Metabolism

**DOI:** 10.1371/journal.pcbi.1002577

**Published:** 2012-06-21

**Authors:** Matthias König, Sascha Bulik, Hermann-Georg Holzhütter

**Affiliations:** Institute of Biochemistry, University Medicine Charité Berlin, Berlin, Germany; University of Virginia, United States of America

## Abstract

Despite the crucial role of the liver in glucose homeostasis, a detailed mathematical model of human hepatic glucose metabolism is lacking so far. Here we present a detailed kinetic model of glycolysis, gluconeogenesis and glycogen metabolism in human hepatocytes integrated with the hormonal control of these pathways by insulin, glucagon and epinephrine. Model simulations are in good agreement with experimental data on (i) the quantitative contributions of glycolysis, gluconeogenesis, and glycogen metabolism to hepatic glucose production and hepatic glucose utilization under varying physiological states. (ii) the time courses of postprandial glycogen storage as well as glycogen depletion in overnight fasting and short term fasting (iii) the switch from net hepatic glucose production under hypoglycemia to net hepatic glucose utilization under hyperglycemia essential for glucose homeostasis (iv) hormone perturbations of hepatic glucose metabolism. Response analysis reveals an extra high capacity of the liver to counteract changes of plasma glucose level below 5 mM (hypoglycemia) and above 7.5 mM (hyperglycemia). Our model may serve as an important module of a whole-body model of human glucose metabolism and as a valuable tool for understanding the role of the liver in glucose homeostasis under normal conditions and in diseases like diabetes or glycogen storage diseases.

## Author Summary

Glucose is an indispensable fuel for all cells and organs, but at the same time leads to problems at high concentrations. As a consequence, blood glucose is controlled in a narrow range to guarantee constant supply and on the other hand avoid damages associated with elevated glucose levels. The liver is the main organ controlling blood glucose by (i) releasing newly synthesized or stored glucose in the blood stream when blood glucose is low (ii) using and storing glucose when blood glucose is elevated. These processes are regulated by hormones, in particular insulin, glucagon and epinephrine. We developed the first detailed kinetic model of this crucial metabolic system integrated with its hormonal control and validated the model based on a multitude of experimental data. Our model enables for the first time to simulate hepatic glucose metabolism in depth. Our results show how due to the hormonal control of key enzymes the liver metabolism can be switched between glucose production and utilization. We provide an essential model to analyze glucose regulation in the normal state and diseases associated with defects in glucose homeostasis like diabetes.

## Introduction

The human plasma glucose is kept in a narrow range between minimum values of ∼3 mM after prolonged fasting or extensive muscle activity and maximum values of ∼9 mM reached postprandially [1], [2]. Homoeostasis of plasma glucose is crucial for the organism: Hyperglycemia results in non-enzymatic glycosylation (glycation) and thus loss-of-function of proteins [3], glucose induced oxidative damage [4], [5] and other adverse effects [6], [7]. Hypoglycemia leads to an under-supply of tissues with glucose and is thereby of particular danger for neuronal cells, erythrocytes and fibroblasts, using glucose as dominant or even exclusive energy-delivering fuel under normal physiological conditions.

The liver is a central player in buffering plasma glucose contributing either by net hepatic glucose utilization (HGU) or net hepatic glucose production (HGP) depending on the plasma glucose level exceeding or falling below a critical threshold value (in the following referred to as ‘set point’) of ∼6 mM. Switching between HGP and HGU is therefore a switch between positive (i.e. export of glucose) and negative (i.e. import of glucose) net hepatic glucose balance. This crucial metabolic function of the liver is performed by hepatocytes which exhibit high capacity of glycogenesis, glycogenolysis, glycolysis and gluconeogenesis enabling them to transiently store substantial amounts of glucose as glycogen, to synthesize glucose from lactate, glycerol and glucoplastic amino acids and to convert excess glucose into triglycerides [2], [8], [9].

Glucose homeostasis is controlled by several hormones, with insulin and glucagon being the main counteracting players [1], [10]. Insulin is the only known hormone lowering blood glucose, whereas multiple glucose increasing hormones exist. Glucagon plays the primary role in counter-regulation to hypoglycemia. Epinephrine has a secondary role, becoming critical under impaired glucagon responses, but with reduced effectiveness compared to glucagon [11]–[13]. Other counter-regulatory hormones like cortisol or thyroxin play only a minor role for the liver [10], [12], [13]. The plasma concentrations of insulin, glucagon and epinephrine change as direct response to varying blood glucose [1], [10]. In the liver insulin increases the activity of glucose utilizing pathways (HGU, glycolysis, glycogenesis) and decreases glucose producing pathways (HGP, gluconeogenesis, glycogenolysis), whereas glucagon and epinephrine have contrary effects. Main targets of the gluco-regulatory hormones are key interconvertible enzymes of glucose metabolism like pyruvate kinase or glycogen synthase. The kinetics of the interconvertible enzymes, and consequently also the hepatic glucose metabolism, depends on their phosphorylation state [2], [14] which is altered by the hormones.

Despite the crucial role of the liver for glucose homeostasis, a detailed mathematical model of human glucose metabolism of the liver, indispensable for understanding the hepatic role under normal and impaired conditions like occurring in diabetes, has not been developed yet. Available kinetic models of hepatic glucose metabolism are either minimal models [15]–[18], concentrate on parts of the glucose metabolism [19], or lump reactions [20], [21]. Furthermore, all available models concentrate on the glucose-insulin system, and ignore the crucial insulin antagonists glucagon and epinephrine [15]–[19], or do not include hormonal regulation at all [20], [21]. All mentioned models with exception of [19] ignore the crucial dependency of enzyme kinetics on the respective phosphorylation state of the enzyme completely, an essential mechanism for short term regulation in hepatic glucose metabolism.

Here, we present the first model of hepatic glucose metabolism in molecular detail, which includes the crucial control of hepatic glucose metabolism by insulin, glucagon and epinephrine via changes in phosphorylation state of key enzymes based on a new concept of linking hormonal regulation with metabolism.

## Methods

**Figure 1:**
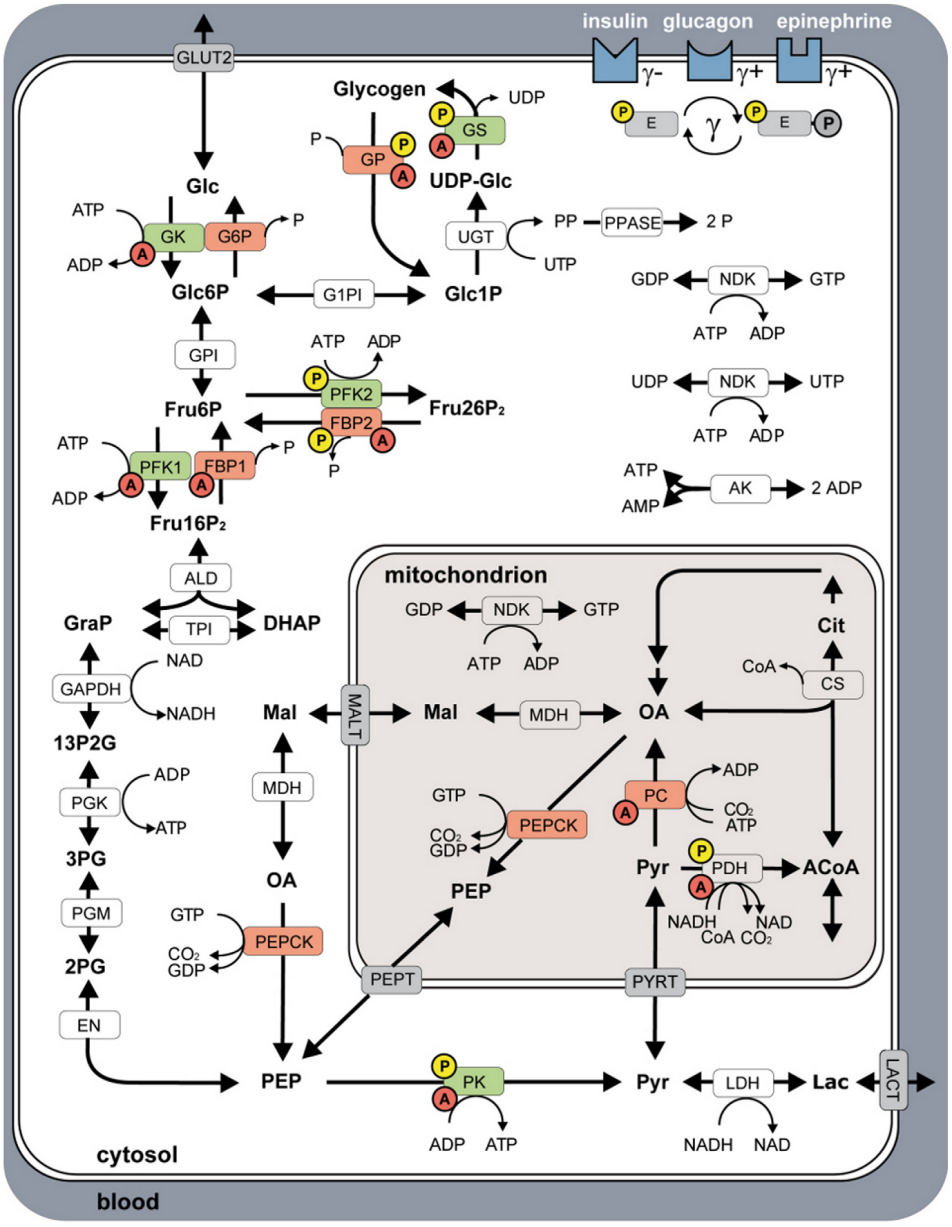
Overview of human hepatocyte model consisting of glycolysis, gluconeogenesis and glycogen metabolism. Metabolic model compartmentalized in blood, cytosol and mitochondrion. Key enzymes of HGP are shown in red, key enzymes of HGU in green. Enzymes regulated via allosteric mechanisms are marked with a red A, interconvertible enzymes with a yellow P. Insulin, glucagon and epinephrine regulate the glucose metabolism by changing the phosphorylation state 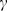 of key interconvertible enzymes, with insulin (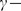
+) decreasing and epinephrine (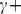
+) and glucagon (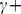) increasing 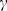. See Methods for additional information. Reactions: (ALD) aldolase, (AK) adenylate kinase, (CS) citrate synthase, (EN) enolase, (FBP1) fructose-1,6 bisphosphatase, (FBP2) fructose-2,6 bisphosphatase, (GAPDH) glyceraldehyde phosphate dehydrogenase, (G1PI) glucose-1 phosphate 1,6-phosphomutase, (G6P) glucose-6 phosphatase, (GK) glucokinase, (GLUT2) glucose transporter 2, (GP) glycogen phosphorylase, (GPI) glucose-6 phosphate isomerase, (GS) glycogen synthase, (LDH) lactate dehydrogenase, (MALT) malate transporter, (MDH) malate dehydrogenase, (NDK) nucleoside diphospate kinase, (PC) pyruvate carboxylase, (PEPCK) phosphoenolpyruvate cyrboxykinase, (PEPT) phosphoenolpyruvate transporter, (PDH) pyruvate dehydrogenase, (PFK1) phosphofructokinase 1, (PFK2) phosphofructokinase 2, (PGK) phosphoglycerate kinase, (PGM) 3-phosphoglycerate mutase, (PK) pyruvate kinase, (PPASE) pyrophosphate phosphohydrolase, (PYRT) pyruvate transporter, (TPI) triose phosphate isomerase, (UGT) UTP:glucose-1 phosphate uridylyltransferase. Metabolites: (13P2G) 1,3-bisphospho glycerate, (2PG) 2-phospho glycerate, (3PG) 3-phospho glycerate, (ACoA) acetyl-coA, (Cit) citrate, (CoA) coenzyme A, (DHAP) dihydroxyacetone phosphate, (Fru16P2) fructose-1,6 bisphosphate, (Fru26P2) fructose-2,6 bisphosphate, (Fru6P) fructose-6 phosphate, (Glc) glucose, (Glc1P) glucose-1 phosphate, (Glc6P) glucose-6 phosphate, (GRAP) glyceraldehyde 3-phosphate, (Lac) lactate, (OA) oxalacetate, (Mal) malate, (P) phosphate, (PEP) phosphoenolpyruvate, (PP) pyrophosphate, (Pyr) pyruvate, (UDP-Glc) UDP-glucose.

The model of human hepatic glucose metabolism (Figure 1) consists of 49 localized metabolites (Text S1) and 36 reactions (Text S2) compartmentalized into cytosol, mitochondrion and blood. Abbreviations of metabolites and reactions are defined in the legend of Figure 1. All reactions and transporters are modeled with individual kinetics (Text S3) with parameters from literature research or by fitting to experimental metabolite and flux data (Equation 1, Dataset S1). An annotated SBML of the model is provided in the supplement (Dataset S2). The model includes regulation via allosteric mechanisms (Text S4) and regulation by hormones via phosphorylation and dephosphorylation of interconvertible enzymes (Text S5). Enzymes regulated by allosteric mechanisms are GK, GS, GP, PFK1, PK, PC, PDH, FBP1 and FBP2. Enzymes regulated via changes in phosphorylation state are GS, GP, PFK2, FBP2, PK and PDH. All model fluxes are given in 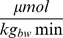 (bw = bodyweight).

### Kinetic parameters

The detailed kinetic equations for the reactions and transporters (Text S3) are specific for human liver and based on extensive literature research. All kinetic parameters except the maximal velocities 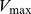 are based on literature data with references given in the respective rate equation. All 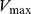 values in the model were determined by fitting simulated model fluxes 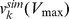 to experimentally determined fluxes 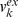 and simulated model metabolite concentrations 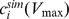 to experimentally measured concentrations 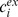.

The fit procedure minimizes the sum of quadratic relative differences in fluxes and concentrations, divided by the total number of experimental fluxes 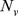 or respectively the total number of experimental concentrations 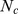 (Equation 1). Relative differences were used to avoid a domination of the optimization by large absolute values and to have dimensionless quantities. The contributions of fluxes and concentrations to least-square fit function were weighed equally (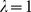) assigning same relevance to fluxes and concentrations.
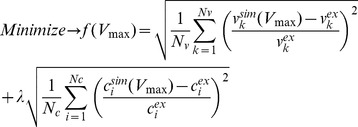
(1)The experimental flux data 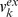 was human specific (Dataset S1), the metabolite concentrations 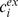 were taken from human and rat liver and are given in (Text S1, Dataset S1). Equation 1 was minimized using the MATLAB® Optimization Toolbox (constraint nonlinear optimization) and resulted in a final value of 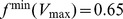, indicating that the overall remaining relative deviations of theoretical values from experimental ones were lower than a factor of 2. The fitted 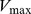 values are given in Text S2.

### Integration of differential equations

For the time course and steady state simulations the differential equation system (Text S3) was integrated with a variable-order solver for stiff problems based on numerical differentiation formulas with absolute integration tolerance 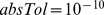 and relative integration tolerance 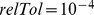 (ode15s MATLAB® R2011a, MathWorks). Initial concentrations for all simulations are given in Text S1. Variation in blood glucose and glycogen are given in the respective figure legends. The external concentrations of blood glucose and lactate were kept at fixed values in all simulations as their time evolution in the blood depends on the metabolic activity of various organs not considered in this model. The cellular redox state (given by the concentrations of NAD and NADH) and cellular energy state (given by the concentrations of the adenine nucleotides) were kept constant during all simulations.

Steady state solutions are defined as states with absolute changes in every concentration smaller than 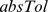 for a time interval 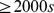. Steady state solutions were tested to be stable against small changes in initial concentrations (1%). All conservation entities were tested to be constant within the tolerances over the integration.

### Hormonal regulation (GHR)

**Figure 2:**
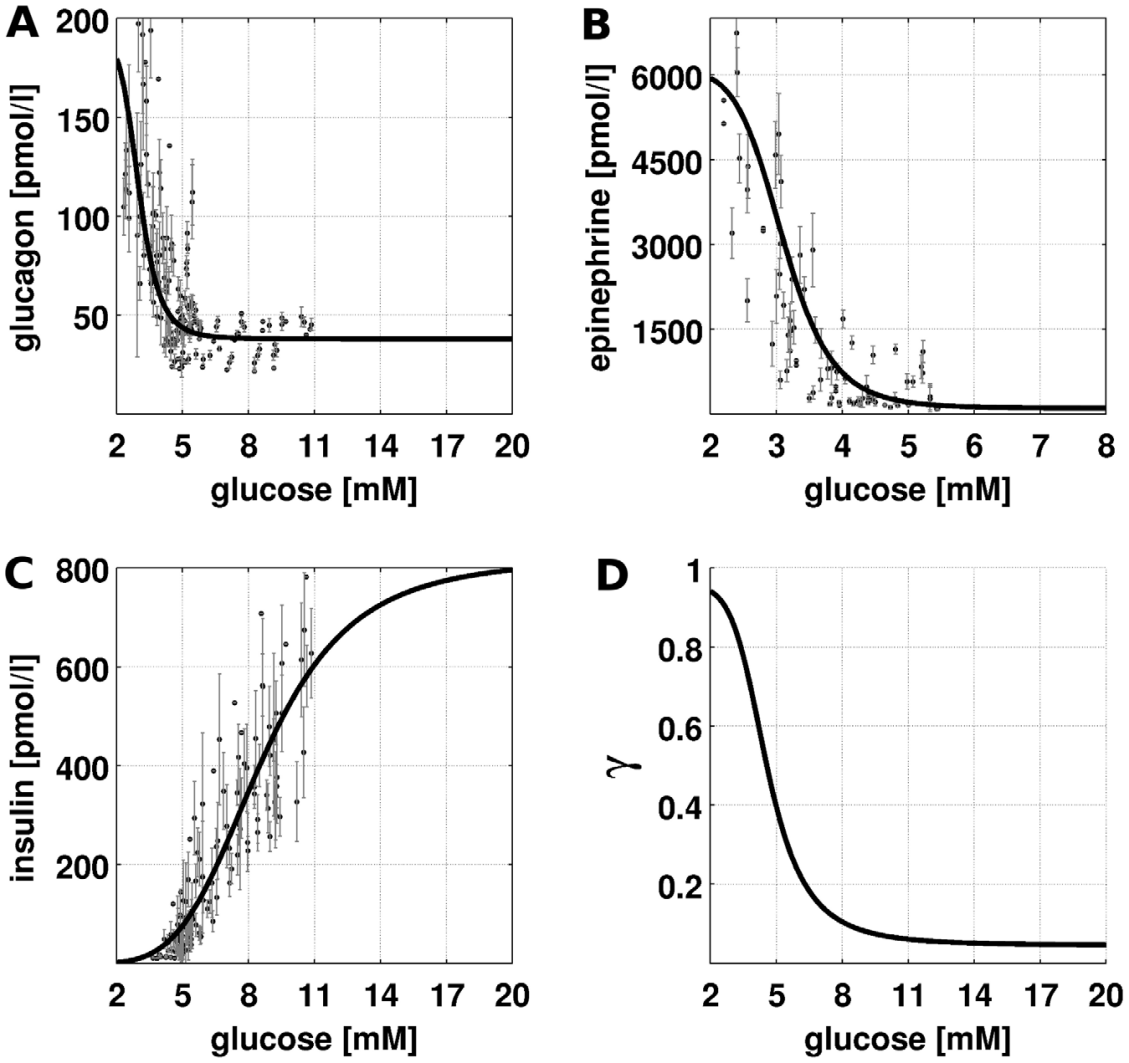
Glucose-Hormone Responses (GHR) of insulin, glucagon and epinephrine and phosphorylation state 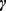. Experimental data from multiple studies based on oral glucose tolerance tests and hypoglycemic, hyperinsulinemic clamp studies and the corresponding fitted GHR curves. Parameters for the GHR curves in Table 1 and experimental data in Dataset S1. (A) Glucagon GHR. Glucagon increases with decreasing blood glucose from a basal concentration of 37.9 pmol/l to a maximal concentration of 190.0 pmol/l with the inflection point at 3.01 mM (B) Epinephrine GHR. Epinephrine increases with decreasing glucose from a basal concentration of 100 pmol/l to a maximal concentration of 6090 pmol/l with the inflection point at 3.1 mM (C) Insulin GHR. Insulin increases with increasing glucose from a basal concentration of 0 pmol/l to a maximal concentration of 818.9 pmol/l with the inflection point at 8.6 mM (D) Resulting phosphorylation state 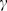 (Equation 5) based on the GHRs for insulin, glucagon and epinephrine decreasing with increasing glucose from phosphorylated to dephosphorylated.

As the release of hormones and their elimination from the plasma are not part of the model, the relationships between plasma glucose level and hormone levels (Figure 2) are described by phenomenological functions called glucose-hormone responses (GHR). The GHRs are sigmoid functions ranging between basal hormone concentration h_base_ and maximal hormone concentration h_max_, monotonically increasing with increasing blood glucose for insulin (Equation 2), monotonically decreasing for glucagon and epinephrine (Equation 3). The GHRs were determined by least-square fit to oral glucose tolerance tests and hypoglycemic, hyperinsulinemic clamp studies (Table 1, Dataset S1). The parameters correspond to 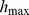, 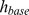, the inflection point 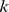 and the Hill coefficient 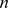 which determines the steepness of the sigmoidal hormone response.
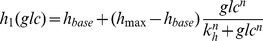
(2)

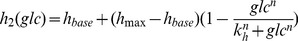
(3)Experimental data and standard deviations (Dataset S1) were extracted from figures, tables and supplemental information. Data points correspond to mean data for multiple subjects from the studies. No data points were omitted.

**Table 1:** Fit functions and parameters for the insulin, glucagon and epinephrine GHR.

Hormone	f	max	base	k	n	Experimental Data
		[pmol/l]	[pmol/l]	[mM]		
insulin	h_1_	818.9	0	8.6	4.2	[1], [28], [35]–[41]
glucagon	h_2_	190	37.9	3.01	6.4	[1], [28], [35], [37], [38], [40]–[46]
epinephrine	h_2_	6090	100	3.1	8.4	[40], [42]–[47]

### Phosphorylation state (γ) of interconvertible enzymes

The short-term effects of insulin, glucagon and epinephrine result from changes in the phosphorylation state of key interconvertible enzymes of glucose metabolism, namely GS, GP, PFK2, FBP2, PK and PDH. The interconvertible enzymes exhibit different kinetic properties in the phosphorylated state (P) and dephosphorylated state (DP), thus carrying different fluxes in the phosphorylated state (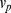) and dephosphorylated state (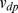). The resulting effective kinetics 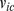 (Equation 4) is the linear combination of 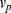 and 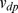 dependent on the phosphorylation state 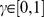, with 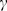 being the phosphorylated and 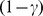 the dephosphorylated fraction of the enzyme.
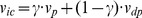
(4)The phosphorylation state 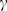 is a phenomenological function of insulin, glucagon and epinephrine (Equation 5). Insulin decreases 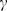, whereas glucagon and epinephrine increase 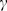. Epinephrine acts as a backup system for glucagon with reduced effectiveness 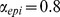. Only the currently dominating hormone (glucagon or epinephrine) was taken into account (max). The hormonal dependencies on the phosphorylation state were modeled by a Michaelis-Menten like hyperbolic function with half-saturation at 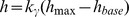 whereby the saturation parameter was set to 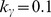 for all hormones. The maximal basal hormone concentrations of the three hormones (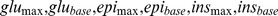) result from the respective GHR curves and are given in Table 1.
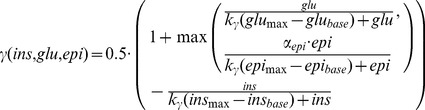
(5)We assumed that for given hormone concentrations the fraction 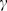 of all interconvertible enzymes is equal.

### Hepatic glucose production, gluconeogenesis and glycogenolysis

Experimental data (Table 2, Dataset S1) was extracted from figures and tables of 25 independent studies with different tracer methods. See [2] for review and Table 2 for detailed references, used methods and experimental data. Every data point is the mean for multiple subjects from one of the studies. No data points were omitted.

**Table 2:** Experimental data for HGP, gluconeogenesis (GNG), glycogenolysis (GLY).

Method	Reference	Time	HGP	GNG	GLY	GNG/HGP
		[h]	[µmol/kg(bw)/min]	[µmol/kg(bw)/min]	[µmol/kg(bw)/min]	[%]
^14^C-acetate	[48]	66	7.56	7.39	0.34	97
^13^C-NMR	[22]	42–64	8.7	8.3	0.3	96
^13^C-glycerol MIDA	[49]	60	7.87	7.71	1.58	98
^13^C-glucose MID	[50]	40	9.8	9.1	1.1	92
^2^H_2_O	[51]	42	-	-	-	93
^2^H_2_O	[52]	40	7.93	7.13	0.8	90
^14^C -acetate	[48]	14	12.5	3.6	9	28
^14^C -bicarbonate	[53]	10–12	7.15	2.2	4.9	31
^14^C -bicarbonate	[54]	12–14	8.3	2.6	5.5	31
^14^C -glucose	[55]	12	8.8	4.5	4.3	51
^13^C-NMR	[22]	22	12.2	7.9	4.3	64
^13^C-NMR	[23]	23	8.9	6.1	2.8	70
^13^C-glycerol MIDA	[49]	11	12.1	5.9	6.2	49
^13^C-glycerol	[56]	10	11.7	4.9	6.8	41
^13^C-glucose MID	[50]	12	12.9	5.3	7.7	41
^13^C-glucose	[57]	-	13.1	7.4	5.7	56
^13^C-glucose (a)	[58]	18	8.5	5.9	2.6	59
^13^C-glucose (b)	[58]	18	8.5	3.7	4.8	44
^13^C-glucose	[59]	12	12.9	5.3	7.6	41
^13^C-glucose	[59]	16	11.5	6.6	4.8	57
^13^C-glucose	[59]	20	10	7.1	2.9	71
^2^H_2_O	[51]	14	10.2	5.5	4.7	54
^2^H_2_O	[51]	22	8.6	5.5	3.1	64
^2^H_2_O	[60]	16	10	5.5	4.5	55
^2^H_2_O	[60]	20	9	5.4	3.6	60
^2^H_2_O	[60]	24	8.5	5.2	3.3	61
^2^H_2_O	[61]	10	11.4	5.5	5.9	48
^2^H_2_O	[62]	16	12.2	6.7	5.5	55
^2^H_2_O	[63]	15	12.4	5.6	6.7	45
^2^H_2_O	[56]	10	12.2	7.4	3.8	60
^2^H_2_O	[64]	11	10.4	5.8	4.65	55
^2^H_2_O	[65]	16	9.8	5	4.8	51
^2^H_2_O	[66]	15	17.7	12.7	5	71
^2^H_2_O	[67]	16	17.5	8.9	8.2	51
^2^H_2_O	[68]	17	11.9	4.6	7.3	38

### Glycogenolysis and glycogen synthesis

Experimental data (Dataset S1) was extracted from figures and tables from [22] (individual data) and [23] (mean person data with STD) for the glycogenolysis and from [8], [24], [25] (mean person data) for the glycogen synthesis. The only difference between model simulations in states of either net glycogen synthesis or net glycogenolysis simulations are differences in the initial glycogen and blood glucose concentrations. Experimental data for the mixed meal and hyperglycemic, hyperinsulinemic clamp simulations were extracted from [26] using the experimental time courses of glucose as model input. Insulin and glucagon time courses were predicted with the respective hormone dose response curves (Equation 2 and Equation 3). Simulations were started the from initial glycogen concentrations reported in the experiments.

### Role of insulin and glucagon for HGP

Experimental data (Dataset S1) were extracted from figures and tables from [27]. Basal hormone concentrations of dogs and humans differ due to difference in human and dog physiology. Therefore, in model simulations of the human liver typical insulin and glucagon concentrations for the basal glucose production rate in humans were used (insulin 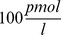 and glucagon 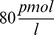) and concentration changes for the hormones expressed relative to these basal values Changes in blood glucose were calculated from the simulated HGP, for a mean bodyweight of 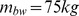, blood volume of 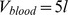 and whole-body basal glucose utilization rate of 
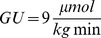
. In Simulation 1 no experimental data was taken into account, but only a drop in the respective hormones during the infusion period (135–200 min) assumed. In Simulation 2 the experimentally observed changes in hormones and GU relative to basal levels (mean of values during the pre-infusion period) were used.

### Hepatic glucose response coefficient (HGRC)

The HGRC defined by Equation 6 measures the change in net hepatic glucose balance defined through the net rate of glucose exchange between hepatocytes and the blood (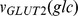) elicited by a small change in blood glucose concentration. In model simulations, the HGRC was approximated by a difference quotient and numerically calculated from the steady state GLUT2 fluxes. For 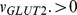 the HRGC corresponds to the changes in HGU, for 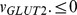 to the change in HGP.
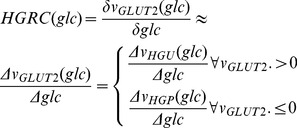
(6)


### Coupling of metabolic pathways to the TCA cycle

The reactions for citrate efflux, oxaloacetate influx and acetyl-CoA flux were included in the model, to test the model under various TCA cycle loads and changes in acetyl-CoA demand and production being a necessary condition in the model development of a functioning model of hepatic glucose metabolism under typical physiological TCA cycle loads. The malate-aspartate shuttle (MALT) including cytosolic and mitochondrial malate dehydrogenase (MDH) was not modeled in detail. Thus, the rate of the cytosolic isoform of the PEPCK was put to directly depend on mitochondrial oxaloacetate concentration. For the actual simulations these boundary fluxes where set to zero.

### Metabolic Control Analysis (MCA)

To characterize the regulatory importance of various reactions in different states of hepatic glucose metabolism we used metabolic flux response coefficients 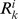 (Equation 7), describing how the flux rate of an arbitrary reaction 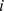 changes in response to a small change in the maximal activity 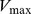 of an arbitrary reaction 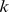.
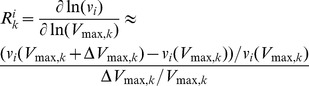
(7)Response coefficients with respect to the maximal activities of all enzymes were calculated at varying values of external glucose and cellular glycogen content for key fluxes of hepatic glucose metabolism (Figure S9): hepatic glucose uptake/release (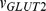), gluconeogenesis/glycolysis (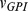) and glycogenolysis/gluconeogenesis (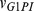).

## Results

**Figure 3:**
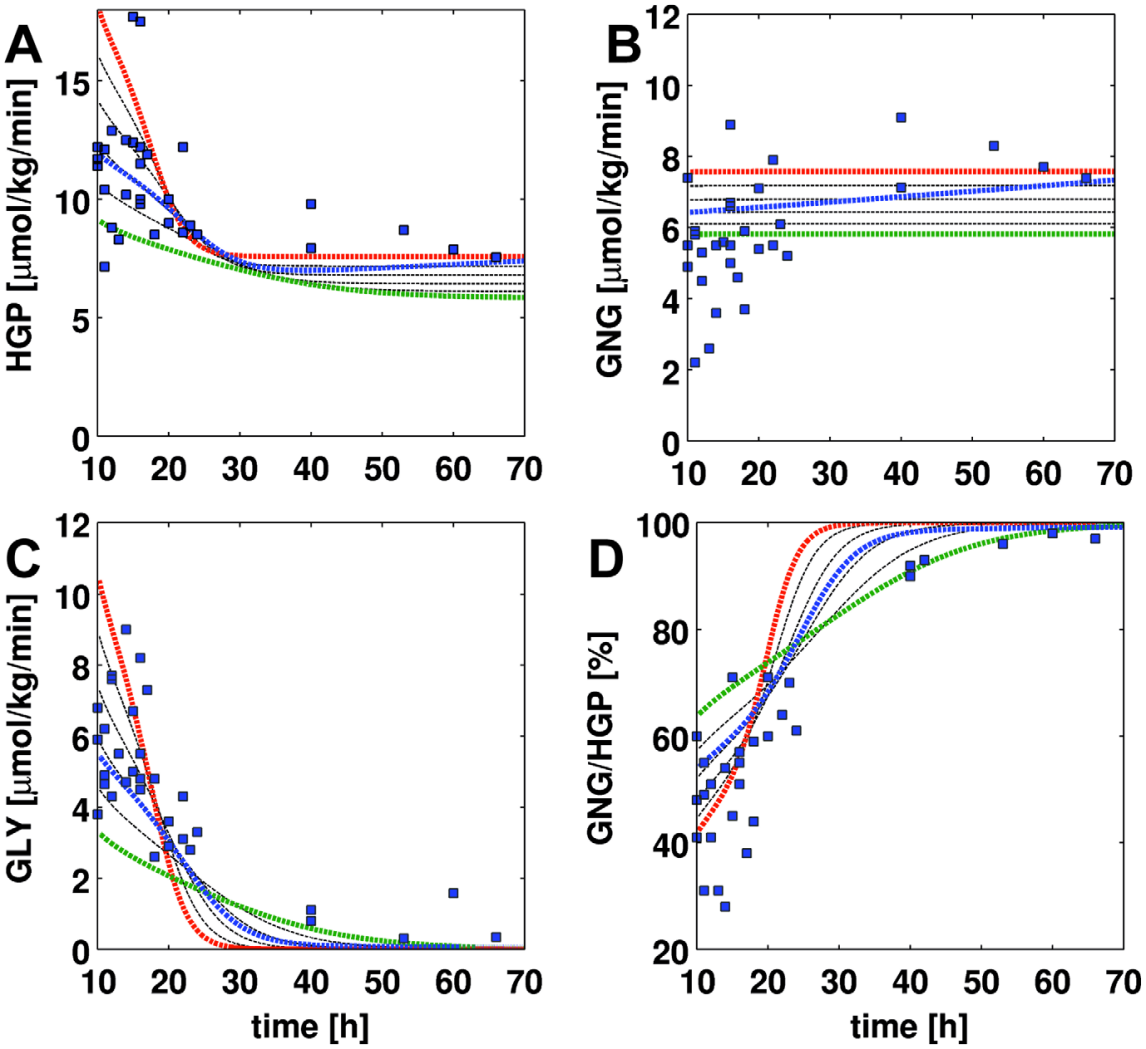
HGP, gluconeogenesis (GNG), glycogenolysis (GLY) and GNG/HGP in short term fasting over 70 h. Experimental data from 25 separately published studies based on a variety of different labeling approaches listed in Table 2 and given in Dataset S1. Simulation time courses (dashed lines) start at t = 10 h with glycogen partially filled glycogen stores (350 mM). Blood glucose is varied between 3.6 mM (red) and 4.6 mM (green) in steps of 0.2 mM. The blue curve corresponds to a situation where the plasma glucose concentration changes over the time of fasting from 4.2 mM to 3.6 mM in 70 h. (A) HGP decreases with increasing fasting time and reaches constant levels after around 25 h. With decreasing blood glucose HGP increases. (B) gluconeogenesis is almost constant over time. With decreasing blood glucose GNG increases (C) glycogenolysis decreases over time with decreasing glycogen. Lower blood glucose results in an initially higher GLY rate (D) GNG/HGP. The relative contribution of gluconeogenesis to the hepatic glucose production increases over time. After 40 h fasting over 90% of the produced glucose comes from de novo synthesis.

**Figure 4:**
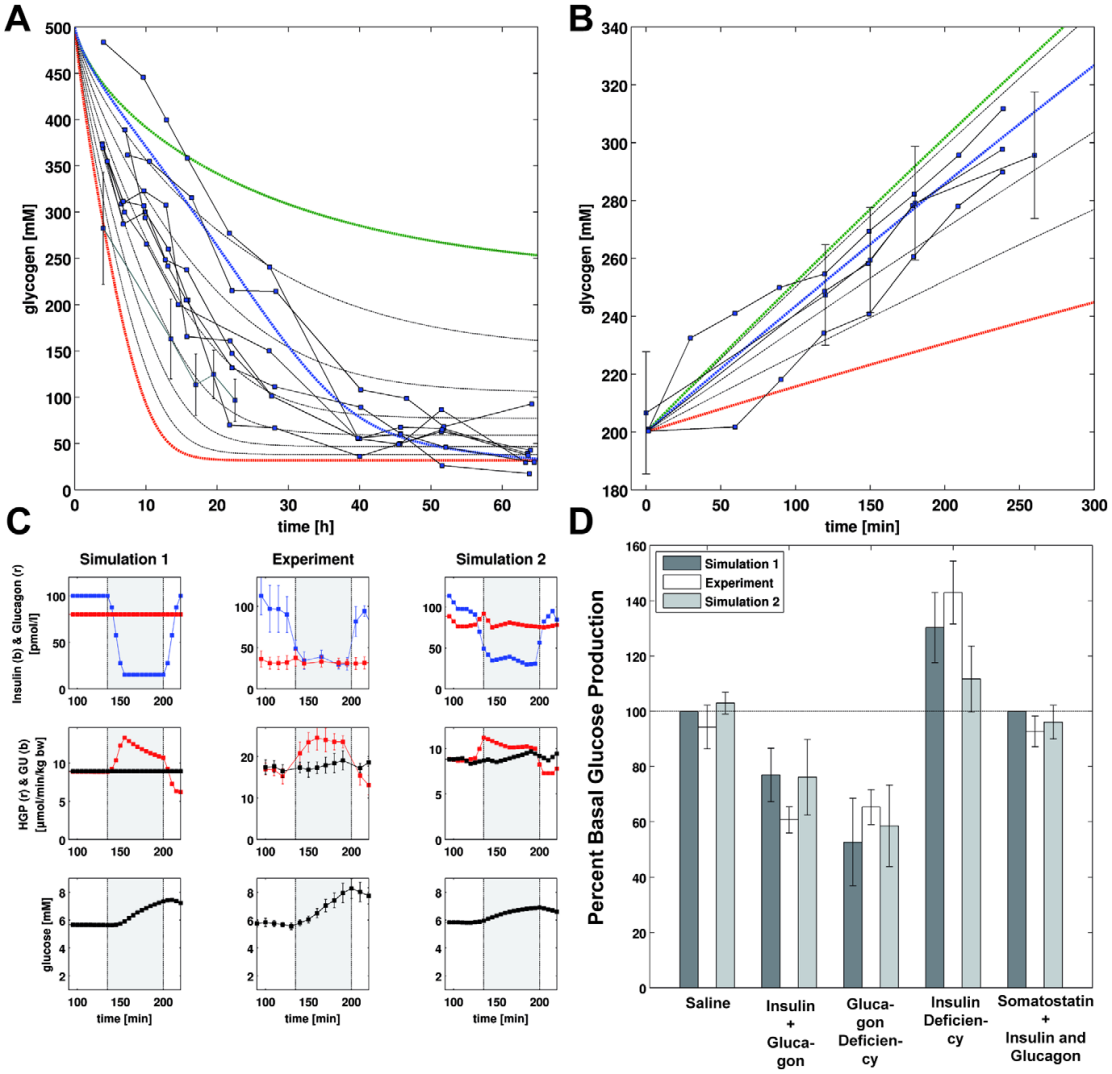
Glycogen metabolism and hormone perturbations. (A) Decrease in glycogen during short term fasting over 60 h. Experimental data from [22] (individual data) and [23] (mean data with STD) given in Dataset S1. Simulation time courses (dashed lines) start from filled glycogen stores (500 mM). Blood glucose is varied between 3.6 mM (red) and 5.0 mM (green) in steps of 0.2 mM (black). The blue curve corresponds to a situation where the plasma glucose concentration changes from initially 5 mM to 3.6 mM in 60 h. Higher blood glucose results in a slower decrease in glycogen. (B) Increase in glycogen after a glucose load. Experimental data from [8], [24], [25] given in Dataset S1. Simulation time courses (dashed lines) start from initial glycogen concentration of 200 mM. Blood glucose concentrations are varied between 5.5 mM (red) and 8.0 mM (green) in steps of 0.5 mM (black). The blue line corresponds to 7 mM. Higher blood glucose results in a faster increase in glycogen after a glucose load. (C) Change in HGP and plasma glucose in insulin deficiency. Insulin (blue) and glucagon (red) time courses for an idealized hormone perturbation (left), the experimental data from [27] (middle), and a simulation taking the experimental profile of hormones and glucose utilization into account are shown in the top row. Middle row depicts the time courses of HGP (red) and whole-body glucose utilization GU (black). Bottom row shows the resulting change in plasma glucose as a consequence of the changes in HGP. Insulin deficiency increases HGP relative to GU resulting in an increase in plasma glucose. After insulin normalization HGP decreases and falls below GU due to the increased blood glucose. (D) Changes in HGP during the infusion period (135–200 min) relative to the pre-infusion period (85–135 min) for the simulations and experiments and the various hormone perturbations. Saline infusion (Figure S1) and somatostatin infusion with insulin and glucagon restauration (Figure S5) have no effect on basal HGP. Insulin and glucagon deficiency (Figure S2) and glucagon deficiency alone (Figure S3) decrease HGP relative to basal values. Insulin deficiency increases basal HGP (Figure 4C, Figure S4).

**Figure 5:**
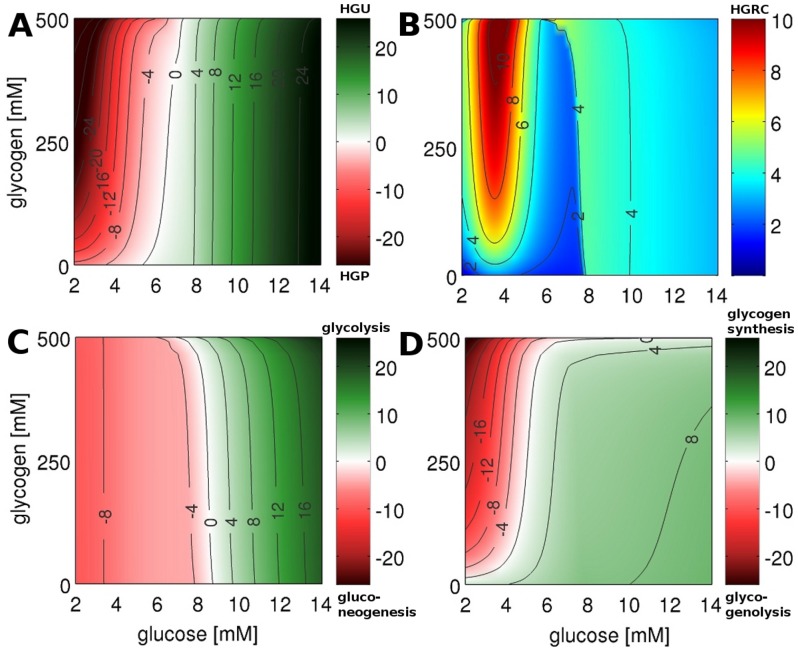
HGP/HGU and HGRC. Steady state solution of time course simulations under constant glycogen and blood glucose concentration (every data points correspond to a single simulation to steady state). Glycogen is varied between 0 and 500 mM in steps of 5 mM, blood glucose is varied between 2 and 14 mM in steps of 0.05 mM. (A) HGP (red)/HGU (green) ([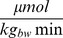]) corresponding to GLUT2 flux. The liver switches between HGP and HGU depending on the blood glucose and glycogen. The set point varies between 5.4 mM for empty and 7.5 mM for completely filled glycogen stores (6.6 mM at glycogen 250 mM). (B) HGRC ([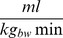]). Response of the liver to changes in the blood glucose. Two main regions of high response exist: A hypoglycemic region below 4 mM, and a hyperglycemic region above 8 mM glucose. (C) gluconeogenesis (red)/glycolysis (green) ([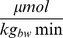]) corresponding to glucose-6p isomerase (GPI) flux. The gluconeogenesis/glycolysis set point varies between 8.8 mM for empty and 7.0 mM for filled glycogen stores. (D) glycogenolysis (red)/glycogenesis (green) ([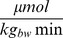]) corresponding to flux through glucose-1p isomerase (G1PI).

We present the first detailed kinetic model of human hepatic glucose metabolism integrated with the hormonal regulation via insulin, glucagon and epinephrine (Figure 1 and Methods) via a new concept of changes in phosphorylation state (Figure 2 and Methods). The model was validated with quantitative and qualitative experimental data from a multitude of studies on hepatic glucose metabolism in overnight fasting, short term fasting and postprandial glycogen storage (Table 3, Figure 3, Figure 4, Dataset S1). In a next step the model was applied to analyze the central role of the liver in glucose homeostasis, especially the hepatic capability (i) to switch between an anabolic glucose mode (HGP) to a catabolic glucose mode (HGU) depending on the blood glucose concentration and hormonal signals (iii) to use glycogen as glucose buffer in this process (iii) to respond to counteracting changes in blood glucose with changes in glucose production (HGP) and glucose utilization, especially in the fasting and postprandial physiological state (Figure 5).

**Table 3:** Comparison of model predictions and experiments.

	Item	Model	Experimental data	References
**A**	HGP at −5.5 mM blood glucose HGU at 8 mM blood glucose	∼4 µmol/kg(bw)/min HGU at 8 mM blood glucose	∼8.5 µmol/min/kg(bw) splanchnic glucose utilization (SGU) at 8 mM glucose (difference between 7 µmol/min/kg (bw) splanchnic glucose production and 15.5 µmol/min/kg (bw) splanchnic glucose uptake at physiological insulin of 300 pmol/l at 8 mM)	[29]
		HGP/HGU set point: 6.6 mM glucose for half-filled glycogen; 7.3 mM glucose for filled glycogen stores;	HGU<SGU due to glucose usage of the gut	[29]
**B**	set point glycogenesis/glycogenolysis	set point at 5.1 mM	set point at ∼5 mM (∼6 h postprandially)	[8], [25]
**C**	rate of glycogenesis and cumulative glycogen content	increase from 250 to 350 mM glycogen at 7 mM (250 to 370 mM glycogen at 8 mM) glucose in 4 h with linear rate of glycogenesis	increase from ∼200 to ∼300 mM glycogen at ∼7–8 mM glucose in 4 h with linear rate of glycogenesis	[8], [24], [25]
**D**	HGP after short term starvation and contributions from gluconeogenesis/glycogenolysis	∼8.5 µmol/kg(bw)/min HGP for short term starvation (20 h at 3 mM glucose) with 95% HGP from gluconeogenesis (5% HGP from glycogenolysis)	7.56–9.8 µmol/kg(bw)/min HGP with 92–97% gluconeogenesis (2–8% HGP from glycogenolysis)	[2], [22], [50], [51].
**E**	glycogen decrease (overnight fast)	decrease in glycogen from filled (500 mM) to half-filled glycogen stores (250 mM) in 16 h at 4.5 mM glucose	decrease in glycogen from almost filled stores to ∼half-filled (200–250 mM) glycogen in around 18–20 h	[22]
		rate of glycogenolysis almost constant and only decreasing at low glycogen concentrations	rate of glycogenolysis almost constant and only decreasing at low glycogen concentrations	[22]
**F**	HGP for overnight fast and contributions from gluconeogenesis/glycogenolysis	∼13.5 µmol/kg(bw)/min HGP at ∼3.8 mM blood glucose with ∼5.5 µmol/kg(bw)/min glycogenolysis (41%) and ∼8 µmol/min/kg(bw) gluconeogenesis (59%)	∼12 µmol/kg(bw)/min HGP with nearly equal contributions of glycogenolysis and gluconeogenesis with ∼6 µmol/kg(bw)/min (50%)	[2]
**G**	glycogenesis via direct and indirect pathway	equal rates of HGU and gluconeogenesis of 4 µmol/kg(bw)/min at 8 mM glucose (equal contributions of direct and indirect pathway)	∼equal amounts of glycogenesis via direct (10 g) and indirect pathway (15 g) after oral glucose load	[69]
**H**	rate of glycogenolysis	∼constant rate of glycogenolysis for partially filled glycogen stores and a decrease in glycogenolysis only for glycogen below ∼150 mM	∼constant rate of glycogenolysis for partially filled glycogen stores and a decrease in glycogenolysis only for low glycogen	[22], [23]

### Hormonal regulation of glucose metabolism

The plasma concentrations of the gluco-regulatory hormones change with changes in plasma glucose concentration. With increasing glucose the insulin level increases (Figure 2C), whereas the levels of glucagon (Figure 2A) and epinephrine (Figure 2B) decrease. Consequently, the phosphorylation state 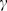 of the interconvertible enzymes (Figure 2D, Equation 5) changes from phosphorylated (94% at 2 mM) to dephosphorylated (5% at 14 mM) with increasing blood glucose. Changes in the phosphorylation state are accompanied by changes of the kinetic properties of the respective enzymes (see Equation 4). Due to the higher activity of the interconvertible enzymes of HGP (GP and FBP2) in the phosphorylated form and the increased activity of the enzymes of HGU (GS, PFK2, PDH, PK) in their dephosphorylated form (Text S5), the hepatic glucose metabolism is shifted from a glucose producing phenotype (HGP) under low glucose concentrations to a glucose consuming phenotype (HGU) at hyperglycemia. This change from an anabolic metabolism of glucose production (HGP) to a catabolic mode of glucose utilization (HGU) is a short term adaptation via changes in the kinetic properties of crucial enzymes of glucose metabolism. By adapting the HGP/HGU, gluconeogenesis/glycolysis and glycogen metabolism to the current hormonal signals and blood glucose concentration, the liver is able to fulfill its important role in glucose homeostasis in a variety of physiological states ranging from hypoglycemic states in overnight and short term fasting to hyperglycemic states postprandial.

### HGP, gluconeogenesis and glycogenolysis in short term fasting

The liver is the main glucose supplier in overnight fasting and short term fasting. The produced hepatic glucose (HGP, Figure 3A) results either from de novo synthesis via gluconeogenesis (GNG, Figure 3B) or from degradation of hepatic glycogen via glycogenolysis (GLY, Figure 3C). The relative contributions of these two processes to HGP change over the time course of fasting with gluconeogenesis becoming more and more important, whereas the share of glycogenolysis to HGP decreases (GNG/HGP, Figure 3D).

The simulations for short term fasting under constant glucose concentrations between 5 mM (green) and 3 mM (red), the normal range of fasting glucose concentration [22], [23], are in agreement with the experimental data (Figure 3, Table 2). Taking into account the gradual decrease in blood glucose concentration during fasting from 5 mM to 3.6 mM (blue), an agreement between simulation and experimental data is observed.

HGP decreases with ongoing fasting to a constant basal rate of 7–8 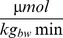 at around 40 h (Table 3D, Table 3F). With increasing blood glucose HGP decreases. Gluconeogenesis rate is constant for given blood glucose concentrations (see [2] for review). Taking into account the gradual decrease in blood glucose over fasting the rate of gluconeogenesis increases gradually (blue). In contrast, glycogenolysis decreases sharply during fasting due to the emptying glycogen stores (see also Figure 4A). As a consequence, the fractional contributions of glycogenolysis and glycogen synthesis to HGP shift from initially equal contribution of both processes to glucose finally completely synthesized de novo. Whereas after an overnight fast (∼10 h) half of the HGP comes from glycogenolysis [2], after 40 h fasting only 10% of HGP result from glycogen, 90% from gluconeogenesis.

### Glycogenolysis in short term fasting

Liver glycogen has an important role as a short term glucose buffer for glucose homeostasis. At low blood glucose concentrations, like in the fasting state or during extensive muscle activity, glucose is released from glycogen (Figure 4A), whereas during periods of high blood glucose like postprandial glycogen is synthesized from glucose (Figure 4B).

Over 40–50 h short term fasting the glycogen stores are emptied. During an overnight fast glycogen is utilized and the resulting glucose from glycogenolysis is exported resulting in half-filled glycogen stores after ∼16 h (Table 3E). The rate of glycogenolysis is almost constant and decreases only at low glycogen concentrations (Table 3E).

The simulations for glycogen depletion under hypoglycemia (Figure 4A) for glucose concentrations between 3.6 mM (red) and 5 mM (green) are in agreement with experimental data [22], [23]. The rate of glycogenolysis depends on the blood glucose concentration (see also Figure 3C). With decreasing blood glucose concentration, the rate of glycogenolysis increases, glycogen stores are emptied faster. As a consequence of the drop in glucose concentration over fasting, simulations at low glucose concentration overestimate the decrease in glycogen, simulations at high glucose concentrations underestimate the depletion. When taking the gradual drop of blood glucose from 5 to 3.6 mM into account (blue), the concordance of simulations experimental data further improved.

### Glycogen synthesis postprandial

Blood glucose levels are elevated postprandial and glycogen is stored via glycogen synthesis (Figure 4B). The simulations for glycogen synthesis under hyperglycemia for different glucose concentrations between 5.5 mM (red) and 8.0 mM (green) (normal range of postprandial glucose concentrations) are in good agreement with the experimental data [8], [24], [25], especially the simulation at 7 mM (blue) representing a normal blood glucose value postprandial. With increasing blood glucose the rate of glycogen synthesis increases and the hepatic glycogen stores are filled faster. For medium filled glycogen stores between 200 and 300 mM the rate of glycogen synthesis is constant for a given blood glucose concentration.

To further evaluate the predictive capacity of our model, we performed time-course simulations of experimentally determined glycogen levels in dogs monitored under conditions of hyperglycemic, hyperinsulinemic clamps and administration of a mixed meal diet [26], as can be seen from the respective figures in the supplement (Figure S1, S2, S3, S4, S5, S6), our model simulation were in good agreement with the measured time courses of insulin, glucagon and glycogen. Of note, in these simulations none of the data was used for the calibration of the model. Moreover, successful simulation of these experiments under conditions where the two hormones insulin and glucagon could be varied independently from each other while in vivo their levels are coupled by the glucose level of the blood demonstrates the validity of the phenomenological glucose-hormone response functions (Equation 2 and 3) and interconversion –versus-hormone function γ (Equation 5) used in our model.

### Role of insulin and glucagon for HGP

To analyze the effects of insulin and glucagon on HGP classical hormone perturbation experiments of hepatic glucose metabolism were simulated [27]. In these experiments a deficiency in either insulin or glucagon or both was achieved by somatostatin infusion, an inhibitor of insulin and glucagon secretion, in combination with hormone replacement infusions. Figure 4C depicts exemplarily the effect of a transient drop of insulin which is characterized by a marked increase in HGP and a consequent rise in plasma glucose concentration. HGP and plasma glucose return to normal after the perturbation. Simulations of other cases (saline control, insulin and glucagon depletion, glucagon depletion and somatostatin in combination with insulin and glucagon restoration) are depicted in Figure S1, S2, S3 and S5, respectively. For all hormone perturbations the time courses of HGP as well as glucose are in good agreement with the experimentally observed changes.

Predicted changes in basal glucose production (HGP) are very similar to the experimentally observed changes (Figure 4C). Insulin and glucagon depletion or glucagon depletion alone reduce the HGP to around 70% of basal values, insulin depletion increases HGP to around 130%. Insulin and glucagon have a dramatic effect on the human hepatic glucose metabolism, with basal glucagon being responsible for about 30% of HGP and basal insulin preventing increased HGP as a consequence of an unrestrained glucagon action [27].

### Glucose homeostasis by the liver at rapidly changing blood glucose levels

The special role of the liver for glucose homeostasis results from the ability to switch between an anabolic glucose producing mode (HGP) to a catabolic glucose utilizing mode (HGU) depending on the blood glucose concentration and hormonal signals. In this process the hepatic metabolism is altered from glucose production via gluconeogenesis and glycogenolysis at hypoglycemia to glucose utilization via glycolysis and glycogen synthesis at hyperglycemia – a short term switch between metabolic pathways occurring in the range of minutes.

To analyze the temporal response of hepatic glucose metabolism to rapid variations in the external glucose concentrations we performed time-dependent simulations with a step-wise constant concentration profile of blood glucose and constant internal glycogen concentrations. (Figure S7 and S8). To evaluate the impact of hormone induced fast changes in the phosphorylation state of key regulatory enzymes on metabolic regulation we performed these simulations also in the absence of this mode of regulation in the glycogen metabolism, i.e. frozen phosphorylation state of glycogen synthase and glycogen phosphorylase and regulation of these enzyme activities only by allosteric effects. We found clear differences in the simulated time-courses (black and red curves in Figure S7 and S8) indicating that hormonal regulation contributes substantially in the rapid adaptation of the network to abrupt changes of blood glucose. In both cases (i.e. presence or absence of hormonal control) the simulation revealed that even after changing the external glucose concentrations abruptly by 2 mM and more a new steady state was reached within several minutes.

### Glucose homeostasis by the liver at slowly changing blood glucose levels

Based on the above finding that the a new metabolic steady-state (except for glycogen) is achieved within a few minutes after abrupt changes of the blood glucose level it appeared justified to treat the metabolic network as being in a quasi-steady state as long blood glucose changes are slow and the glycogen pool is quasi constant.

Hence, the quasi-steady state approximation was applied to simulate HGP/HGU at varying concentrations of blood glucose and cellular glycogen (Figure 5). In these calculations, the external glucose concentration was varied between constant values of 2 to 14 mM and the glycogen concentration kept at constant values between 0 and 500 mM. For each couple of glucose/glycogen values the resulting HGP/HGU (Figure 5A), the contribution of gluconeogenesis/glycolysis to HGP/HGU (Figure 5C), the contribution of glycogenolysis/glycogen synthesis to HGP/HGU (Figure 5D) and the ability of the liver to respond to changes in blood glucose expressed through the response coefficient HGRC (Figure 5B) in this quasi steady-state where analyzed. The fluxes depicted Figure 5 for a given constant glucose and glycogen concentrations are the reached steady state fluxes for this glucose and glycogen concentrations.

The hepatic glucose metabolism switches between anabolic HGP and catabolic HGU at blood glucose concentrations of 6.6 mM for half-filled glycogen stores (Figure 5A). The set point above the normal glucose concentration of around 5–5.5 mM [1], [28] is in line with the role of the liver as glucose producer under normoglycemic conditions [1], [29]. The liver contributes to glucose homeostasis by exporting glucose below the HGP/HGU set point (red) and importing glucose above this threshold (green) (Table 3A). With increasing blood glucose levels, HGP decreases and HGU increases, in accordance with reported suppression of glucose production and increase in HGU with increasing postprandial glucose level [8]. Glycogen has almost no influence on HGU whereas for low blood glucose concentrations HGP strongly depends on glycogen due to increased contribution of glycogenolysis to HGP with increasing glycogen content (Figure 5D). For low glucose concentrations the increase in glycogenolysis with increasing glycogen is strongest for low glycogen levels, more moderate for glycogen concentrations above 200 mM. HGP is maximal for filled glycogen store and low blood glucose.

The switch between gluconeogenesis and glycolysis occurs at 8.5 mM glucose for half-filled glycogen stores (Figure 5C). Gluconeogenesis and glycolysis rate are mainly determined by the prevalent blood glucose and only marginally depend on glycogen. Below 8 mM blood glucose gluconeogenesis is remarkably constant [2] (see also Figure 3B).

For plasma glucose levels below 5.1 mM glucose is released from glycogen stores via glycogenolysis, above 5.1 mM glycogen is synthesized for half-filled glycogen stores (Figure 5D, Table 3B) with rates of glycogenesis and cumulative glycogen as reported (Table 3C). In contrast to gluconeogenesis/glycolysis, glycogen metabolism is markedly affected by the glycogen content. Glycogenolysis is almost constant for partially filled glycogen stores and decreases only for low glycogen concentrations (Table 3H). Glycogenesis increases with increasing glucose concentration (Figure 5D), which is in accordance with the view that blood glucose determines the maximal rate of glycogenesis [8].

Most interestingly the switching behavior for glycogenesis/glycogenolysis and glycolysis/gluconeogenesis is very distinct, with different set points and different dependencies on glucose. Gluconeogenesis is almost constant for low blood glucose and glycolysis increases with increasing glucose. In contrast glycogenolysis is almost constant for high blood glucose and increases with decreasing blood glucose at low blood glucose. In combination of the two processes a HGP/HGU output of the liver is generated with a set point at normal blood glucose concentrations which is able to react over the whole range of physiological glucose concentrations (Figure 5A).

### Direct and indirect glycogen synthesis

As a consequence of the different set points for gluconeogenesis/glycolysis and glycogenesis/glycogenolysis, hepatic glycogen can be synthesized via two alternative pathways: the direct pathway, in which glucose taken from the blood (HGU) is directly stored as glycogen above the gluconeogenesis/glycolysis set point of ∼8.5–8.8 mM; the indirect pathway, in which glucose synthesized via gluconeogenesis, is stored as glycogen for blood glucose concentrations between 5 mM and the HGP/HGU set point of ∼6.6–7 mM; In the intermediate region between the set points of HGP/HGU and gluconeogenesis/glycolysis glycogen is synthesized via the direct and indirect pathway with varying contributions [8] (Table 3G).

### Response coefficients

The effects of changes of 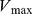 values on HGP/HGU, gluconeogenesis/glycolysis and glycogenolysis/gluconeogenesis were analyzed via response coefficients [30] (Figure S9, Equation 7). The important findings of this analysis are as follows: (i) The effect of changes in the 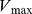 is strongly dependent on the external concentration of glucose and to a much lesser extent on the glycogen level. (ii) Under hypoglycemic conditions main control of hepatic glucose metabolism is exerted by a completely different set of reactions than in hyperglycemia. (iii) The response of the opposing pathway couples gluconeogenesis/glycolysis is very different to the glycogenolysis/gluconeogenesis. (iv) A small group of enzymes catalyzing irreversible reaction steps (GK, G6PASE, GP, PFK1, FBPASE1, PFK2, and FBPASE2) have a significant influence on the hepatic glucose metabolism whereas the majority of enzymes have only marginal effects. This control analysis is of particular interest for gene expression studies of hepatic glucose metabolism like for instance studies of circadian or feeding induced changes in hepatic expression [31].

### The liver – a glucose homeostate (HGRC)

The hepatic counter-regulatory capacity to changes in blood glucose was evaluated using the HGRC (Equation 6, Figure 5B), a measurement of the ability of the liver to react to changes in the blood glucose with changes in HGP or HGU, respectively. Our simulations showed that the liver is able to respond over the whole range of physiological blood glucose concentrations with 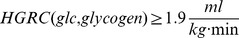 being the lower limit, i.e. a change of 0.01 mM in blood glucose results in a change of HGP/HGU of at least 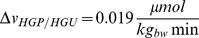. Intriguingly, our analysis suggest the counter-regulatory response of the liver to variations in the external glucose concentration to be particularly pronounced around the HGP/HGU set point at ∼6.6 mM which is flanked by a strong counter-regulatory response to hypoglycemia and a weaker response to elevated blood glucose levels. The strong counter-regulation to a decrease of blood glucose below 5 mM results in an effective increase in HGP whereby the rise of HGP depends on the glycogen content, with most effective counter-regulation for filled glycogen stores. The liver counteracts falling blood glucose levels to avoid hypoglycemia with a strong response. Furthermore, an increased response to elevated blood glucose levels of above 7.5 mM is seen enabling the hepatocyte to react efficiently to elevated blood glucose levels as occurring postprandially (6–10 mM). The hepatic glucose metabolism has ideal regulatory properties to react to the typical physiological challenges to glucose homeostasis: counter regulation to hypoglycemia in fasting and under extensive muscle activity and counter regulation to postprandial increase in blood glucose.

## Discussion

We present the first detailed model of human hepatic glucose metabolism integrating hormonal regulation by insulin, glucagon and epinephrine based on a novel concept to couple the level of these hormones with the phosphorylation state of interconvertible enzymes. This model enables for the first time the analysis of the hepatic carbohydrate metabolism at molecular level, including hormonal regulation. Furthermore, we provide a novel method to integrate hormonal signals with metabolism based on changes in phosphorylation state.

Model simulations are in good agreement with experimental data from a multitude of studies by different laboratories, researchers and methods. We want to emphasize, that the model was not fitted to single study data, but instead, data from a multitude of different studies covering various aspects of glucose metabolism were used. Thereby, the fundamental properties of human hepatic glucose metabolism and not individual study properties could be captured. Remarkably, the agreement of model simulations with numerous experimental and clinical findings was achieved without any re-fitting of model parameters and under neglect of other gluconeogenic substrates than lactate and regulatory phenomena on slow timescales as insulin-dependent changes in the expression level of metabolic enzymes. The model clearly underlines the importance of short term regulation of metabolism by interconvertible enzymes, being able to adapt hepatic metabolism to hormonal signals and glucose levels, and in this process being able to switch between anabolic and catabolic modes even within metabolic pathways.

We simulated the response of the human liver to changes in blood glucose under varying glycogen concentrations and assessed the contributions of glycogen metabolism and glycolysis/gluconeogenesis to HGP/HGU. Thereby we provide essential data for the understanding of the role of the liver in glucose homeostasis, which is not accessible experimentally. The underlying metabolic network reaches quasi-steady state within minutes after perturbations in plasma glucose (see Figure S7 and Figure S8). The dynamics of hepatic glucose metabolism is therefore mainly determined by the depletion/filling of the glycogen stores and the external glucose concentrations under normal conditions. As a consequence, the quasi-steady state system responses, shown for the key fluxes of the glucose metabolism in Figure 5, provide a good approximation of the state of hepatic glucose metabolism at given concentrations of blood glucose and temporary filling state of the glycogen store.

Furthermore, we analyzed the hepatic response to changes in blood glucose over the whole physiological range of blood glucose concentrations (3–11 mM), thereby integrating the available experimental data from the research field of hypoglycemia (accessible with hypoglycemic, hyperinsulinemic clamps <5.5 mM) and of elevated glucose concentrations (accessible with oral glucose tolerance tests OGTT>5 mM).

A model is always an abstraction of reality describing a certain subset of biological phenomena. The underlining assumptions are crucial for to understand the range of application of the model and the limitations. Main model assumptions and simplifications are (i) usage of phenomenological functions to incorporate hormone-induced signal transduction, (ii) a constant cellular redox- and energy status (iii) modeling of an ‘average’ hepatocyte, i.e. neglecting metabolic zonation of hepatocytes along the sinusoid [32] and (iv) no inclusion of changes in the gene expression of metabolic enzymes.

(i) A crucial part of this model is the phenomenological description of signal transduction (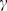), which takes into account a substantial body of qualitative knowledge about the effects of the individual hormones on hepatic glucose metabolism. Due to the lack of quantitative experimental data on signaling processes as, for example, concentrations, activities and phosphorylation states of key kinases and phosphatases at varying concentrations of glucose, insulin, glucagon and combinations of these factors, a more detailed description was not possible. The model reproduces the experimentally observed dependency of glucose metabolism on the hormones. Especially, the reproduction of the classical insulin and glucagon perturbation experiments of [27], performed as independent validation without taking into account in the modeling process, underlines the validity of such a simplified treatment of the signaling network in the case of the hepatic glucose metabolism. Throughout the model the phosphorylation state of all regulatory enzymes is the same at given concentration of glucagon and insulin. This simplification could be the reason why the activation of the glycogen synthase was overestimated in mixed meal and clamp simulations. A second simplification was that the fraction (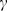) of phosphorylated inter-convertible enzymes follow instantaneously the hormone concentrations in the plasma although signal transduction occurs in the range of some minutes [33]. Including time-dependent changes of proteins involved in the cAMP-dependent signaling cascade in a later and more advanced version of the model should result in slightly different time-courses of fluxes and metabolite concentrations elicited by abrupt changes of external metabolite- and hormone concentrations. However, the main results of this study refer to the normal physiological situation where significant changes of the blood glucose level occur in a time window of hours (Figure 3, 4 and 5) with the metabolic network always being in quasi-steady state.

(ii) The model is limited to physiological states of the liver where changes in the energy- and redox state can be neglected. Normally, the hepatic energy state is decoupled from the hepatic glucose metabolism, with 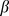-oxidation of fatty acids providing ATP and reduced redox equivalents (NADH). Consequently, the model is not able to simulate conditions where this assumption of energy decoupling is not valid, for example, under hypoxic or ischemic conditions.

(iii) The presented model describes the metabolic net behavior of the liver of a hepatocyte averaged over the liver and is therefore able to simulate the net effect of the liver on glucose balance, namely net HGP and net HGU. In reality the liver exhibits a zonated structure with varying capacities of gluconeogenesis and glycolysis of the hepatocytes along the sinusoid [32]. Some of the effects brought about by an ensemble of hepatocytes equipped with differing capacities of glucose metabolism cannot be simulated with a model of a ‘mean’ hepatocyte. For example, the remaining difference between absolute glucose production and net glucose production (Figure S6) are due to the simultaneous presence of hepatocytes with net HGP (peri-portal hepatocytes) and HGU (peri-central hepatocytes).

(iv) Remarkably, the anabolic/catabolic switch was achieved by fast changes in the phosphorylation state and allosteric regulation of key enzymes of glucose metabolism without temporal changes in gene expression, i.e. changes in the protein levels of enzymes. In our opinion, changes in gene expression as observed to occur with different period lengths during the day [31] play no crucial role in short-term glucose homeostasis, a system which has to react to fast changes in the minute range of glucose supply (postprandial) and glucose demand (muscle activity) by an adaptation of metabolism. Changes in gene expression play a role in adapting hepatic metabolism on longer time scales to for example reduce futile cycles in counteracting pathways (like down-regulation of glucokinase and up-regulation of glucose-6 phosphatase under hypoglycemia) or match glucose metabolism to physiological requirements (up-regulating of glucose-6 phosphatase under hypoglycemic conditions to increase gluconeogenesis). The strong effects of such changes on hepatic glucose metabolism could be seen in the analysis of the response coefficients.

Finally, it has to be noted, that our model comprises fairly active futile cycles in the glycogen metabolism, the PFK1/FBPase1 system and between PEPCK and PK. During model building it was tried to minimize these cycles. Reducing and removing individual cycles in the modeling process compromised the ability of the model to switch between glucose consuming and glucose producing pathways, namely HGP/HGU, gluconeogenesis/glycolysis and glycogen synthesis/glycogenolysis. An essential property of the model is the simultaneous activity of glycogen synthase and glycogen phosphorylase and the accompanied futile cycle, necessary to reproduce the experimental time-courses for glycogen synthesis in fasting and postprandially.

Substrate cycling allows the system to react to fast changes in metabolite levels, the concomitant adaptation of key enzymes via phosphorylation or dephosphorylation causes an additional shift of the net flux in the right direction (see Figure S7). Taken together, metabolic regulation via phosphorylation/dephosphorylation of key enzymes in combination with futile cycles plays a key role in our model, in line with the results presented by Xu et al. [34].

Future work will apply the presented model to diseases of glucose homeostasis like diabetes, integrate the model in models of whole-body glucose metabolism and use individual patient data to generate individualized hepatic glucose models and analyze inter-individual differences in glucose metabolism.

## Supporting Information

Dataset S1 **Experimental data used in model building and validation.** Data spreadsheets for model concentrations, the GHR dose response curves, the glycogen time courses in fasting and postprandial, HGP fluxes, and for the simulations reproducing experimental work by Cherrington and Krssak.
(ZIP)

Dataset S2 **Model SBML.** Annotated SBML of the model.
(XML)

Figure S1 **Cherrington1976 Saline.** Figure analog to Figure 4C. (top row) Insulin (blue) and glucagon (red) time courses for an idealized hormone perturbation (left), the experimental data [27] (center), and taking the experimental profile of hormones and glucose utilization into account (right). (middle row) Time course of HGP (red) and whole-body glucose utilization GU (black) for the respective hormone profiles. (bottom row) Resulting changes in plasma glucose as a consequence of the changes in HGP for the respective hormone profiles and HGP/HGU time courses. Experimental data is provided in Dataset S1.
(TIF)

Figure S2 **Cherrington1976 Insulin and glucagon deficiency.** Figure analog to Figure 4C. (top row) Insulin (blue) and glucagon (red) time courses for an idealized hormone perturbation (left), the experimental data [27] (center), and taking the experimental profile of hormones and glucose utilization into account (right). (middle row) Time course of HGP (red) and whole-body glucose utilization GU (black) for the respective hormone profiles. (bottom row) Resulting changes in plasma glucose as a consequence of the changes in HGP for the respective hormone profiles and HGP/HGU time courses. Experimental data is provided in Dataset S1.
(TIF)

Figure S3 **Cherrington1976 Glucagon deficiency.** Figure analog to Figure 4C. (top row) Insulin (blue) and glucagon (red) time courses for an idealized hormone perturbation (left), the experimental data [27] (center), and taking the experimental profile of hormones and glucose utilization into account (right). (middle row) Time course of HGP (red) and whole-body glucose utilization GU (black) for the respective hormone profiles. (bottom row) Resulting changes in plasma glucose as a consequence of the changes in HGP for the respective hormone profiles and HGP/HGU time courses. Experimental data is provided in Dataset S1.
(TIF)

Figure S4 **Cherrington1976 Insulin deficiency.** Figure analog to Figure 4C. (top row) Insulin (blue) and glucagon (red) time courses for an idealized hormone perturbation (left), the experimental data [27] (center), and taking the experimental profile of hormones and glucose utilization into account (right). (middle row) Time course of HGP (red) and whole-body glucose utilization GU (black) for the respective hormone profiles. (bottom row) Resulting changes in plasma glucose as a consequence of the changes in HGP for the respective hormone profiles and HGP/HGU time courses. Experimental data is provided in Dataset S1.
(TIF)

Figure S5 **Cherrington1976 Somatostatin and insulin and glucagon restoration.** Figure analog to Figure 4C. (top row) Insulin (blue) and glucagon (red) time courses for an idealized hormone perturbation (left), the experimental data [27] (center), and taking the experimental profile of hormones and glucose utilization into account (right). (middle row) Time course of HGP (red) and whole-body glucose utilization GU (black) for the respective hormone profiles. (bottom row) Resulting changes in plasma glucose as a consequence of the changes in HGP for the respective hormone profiles and HGP/HGU time courses. Experimental data is provided in Dataset S1.
(TIF)

Figure S6 **Krssak2004 Postprandial hepatic glycogen metabolism.** Time courses of insulin, glucagon, glycogen and endogenous glucose production/net HGP for mixed meal tests (left) and hyperglycemic, hyperinsulinemic clamp tests (right) in Humans. Experimental data from [26] shown as black box with error bars (experimental data extracted from figures and tables). (I) Blue curves are model simulations with experimental glucose, insulin and glucagon time courses as model input without using hormone dose response curves. (II) Red curves model predictions under given glucose time course with predicted insulin and glucagon time courses via the respective hormone dose response curves as model input. In case of identical simulation results for (I) and (II) only the blue curves are shown. Initial glycogen in the mixed meal simulations are set to 275 mM glycogen and 220 mM glycogen in the clamp simulation. Grey error estimations are based on model simulations taking the errors in insulin and glucagon into account. Predicted insulin, glucagon and glycogen time courses are in agreement with the experimentally observed time courses. Predicted time courses of glycogen and EGP are very similar for (I) and (II). The glycogen synthesis rate in the clamp experiment is overestimated by the model. Imperfect simulation of glycogen synthesis in the mixed-meal and clamp simulations may be due to the fact that throughout the model the phosphorylation state of all regulatory enzymes was the same at given concentration of glucagon and insulin. This assumption is a simplification which could be the reason why the activation of the glycogen synthase was overestimated in our simulations. The measured endogenous glucose production (EGP) and the net hepatic glucose production (HGP) are substantially different. The presented model is a model describing the net hepatic output of the liver, not taking into account possible simultaneous production and utilization of glucose within the liver, for instance in periportal and perivenious regions.
(TIF)

Figure S7 **Fast time course simulation: model concentrations.** Time course of model metabolites during perturbations in plasma glucose from initial 5 mM to 7 mM at t = 0 min, from 7 mM to 3 mM glucose at t = 10 min and from 3 mM back to the initial 5 mM glucose at t = 20 min. Respective fluxes are depicted in Figure S8. Normal model simulations are shown in black. After perturbation the model reaches the new quasi-steady state (only slow change in glycogen) within minutes, even after strong perturbations in blood glucose. To analyze the effect of the hormonal regulation on the glycogen metabolism and to distinguish the effects due to fast allosteric regulations from the hormonal regulation, the model was simulated without changing the phosphorylation state of the glycogen synthase (GS) and glycogen phosphorylase (GP) during the glucose perturbations (red model simulations). The reduced glycogen metabolism leads to an accumulation of the glycolytic intermediates during hyperglycemia due to an inability of the system to store the glucose. If both simulations yield equal results only the red curve is visible.
(TIF)

Figure S8 **Fast time course simulation: model fluxes.** Time course of model fluxes during glucose disturbance profiles. The description of the glucose profile and the corresponding concentration time courses are given in Figure S7. Even without hormonal regulation the glycogen system is able to switch direction between glycogen synthesis and glycogenolysis under hypoglycemia as a consequence of the futile cycle in glycogen metabolism. Hormonal regulation supports the inherent switching of directions increasing the flux in the respective directions. GP decreased in hypoglycemia and strongly increased in hypoglycemia.
(TIF)

Figure S9 **Metabolic control analysis of HGP.** Metabolic response coefficients of the HGP/HGU flux (top), gluconeogenesis/glycolysis flux (middle) and glycogenolysis/glycogenesis flux (bottom) for hypoglycemic conditions of 3 mM (left) and hyperglycemic conditions of 8 mM (right) for various glycogen concentrations. Analyzed were the effects of changes in V_max_ values of the enzymes of hepatic glucose metabolism on the steady state fluxes under the given conditions. The main interesting effects are: (i) The key enzymes depend strongly on the glucose concentration, different enzymes change the HGP/HGU, gluconeogenesis/glycolysis and glycogen synthesis/glycogenolysis under hypoglycemia and hyperglycemia. (ii) Different V_max_ show the main response on HGP/HGU, gluconeogenesis/glycolysis and the glycogen metabolism (iii) The interconvertible enzymes show strong responses to changes in the V_max_ values (iv) Only a selected number of enzymes has a strong response, the change in V_max_ of many enzymes results in almost no change in key glucose fluxes.
(TIF)

Text S1 **Overview metabolites.** The full metabolite name, the used short name in the model, the and the respective compartment are depicted. The Initial concentrations with the respective references used for all simulations are either from human or rat liver/hepatocytes. The concentrations are given in more detailed table format in Dataset S1. In the case that no compartment-specific data for mitochondrion was available, the respective metabolite concentrations for cytosol were used. The glycogen concentration is given in glucose equivalents.
(PDF)

Text S2 **Overview reactions and transporters.** The full reaction/transporter name, the used short name in the model, the and the respective compartment are depicted. The rate equations of the kinetic model are given in Text S3. The given references are the references for the kinetic parameters. Furthermore, the fitted V_max_ values used for simulations are given.
(PDF)

Text S3 **Kinetic rate equations of the model.** Detailed rate equations of all processes of the model of human hepatic glucose metabolism.
(PDF)

Text S4 **Overview allosteric regulations.** The respective rate equations are given (Text S3). Allosterically regulated enzymes characteristic for either HGP or HGU are marked respectively. Allosteric regulators can function as activators are inhibitors. The references stating the roles of the allosteric effectors and the parameters in the rate equations are given.
(PDF)

Text S5 **Overview interconvertible enzymes of human hepatic glucose metabolism.** Overview over the alterations in kinetic properties as a consequence of changed phosphorylation state between phosphorylated and dephosphorylated state of key interconvertible enzymes of HGP and HGU.
(PDF)
