# Supplementary figures and images for: Quantifying the Contribution of the Liver to Glucose Homeostasis: A Detailed Kinetic Model of Human Hepatic Glucose Metabolism
Source: PLoS Comput Biol. 2012 Jun 21;8(6):e1002577. doi: 10.1371/journal.pcbi.1002577 (PMC3383054; doi:10.1371/journal.pcbi.1002577)

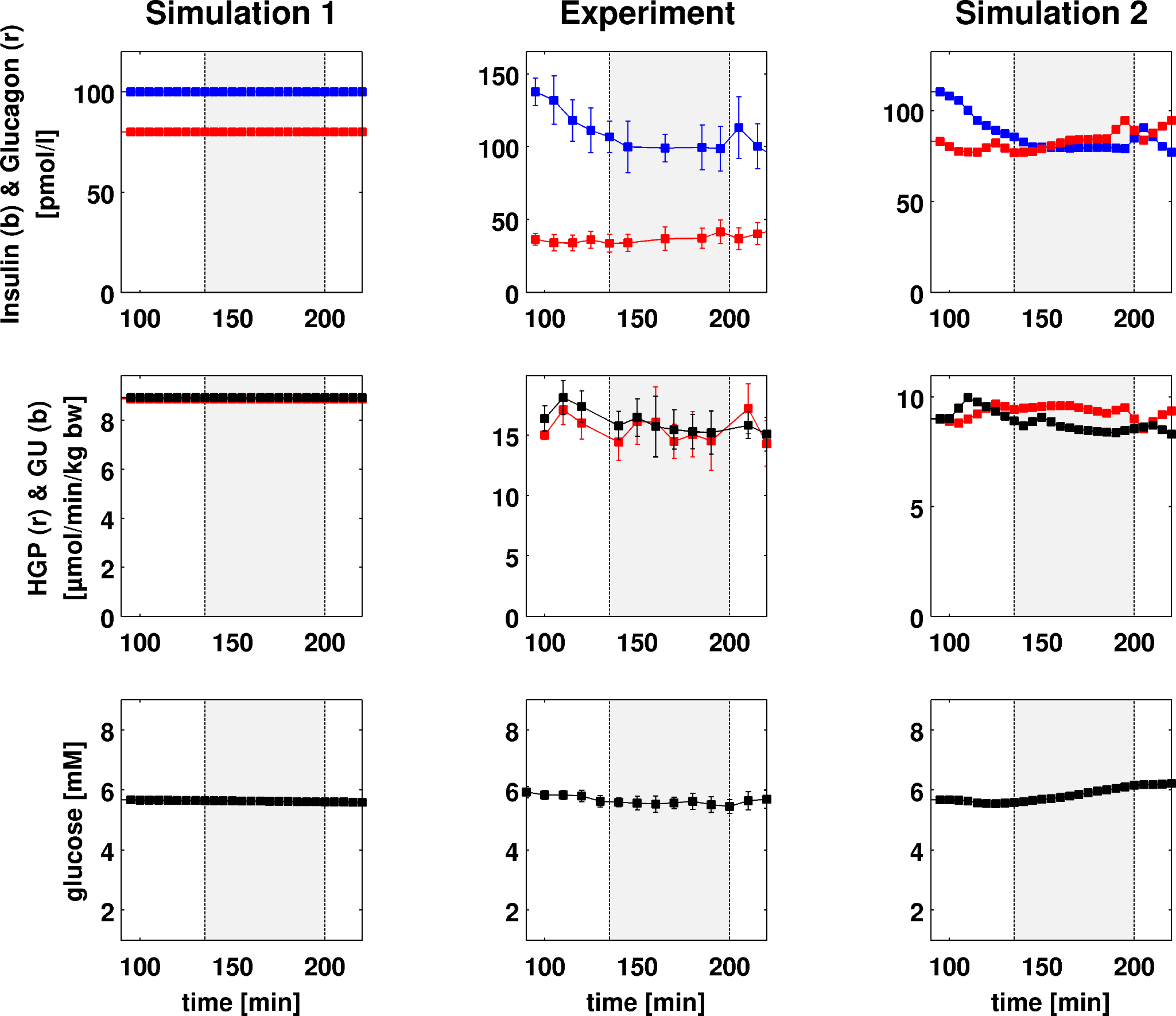

Supplement: Figure S1 — Cherrington1976 Saline. Figure analog to Figure 4C. (top row) Insulin (blue) and glucagon (red) time courses for an idealized hormone perturbation (left), the experimental data [27] (center), and taking the experimental profile of hormones and glucose utilization into account (right). (middle row) Time course of HGP (red) and whole-body glucose utilization GU (black) for the respective hormone profiles. (bottom row) Resulting changes in plasma glucose as a consequence of the changes in HGP for the respective hormone profiles and HGP/HGU time courses. Experimental data is provided in Dataset S1. (TIF) [file pcbi.1002577.s003.tif]

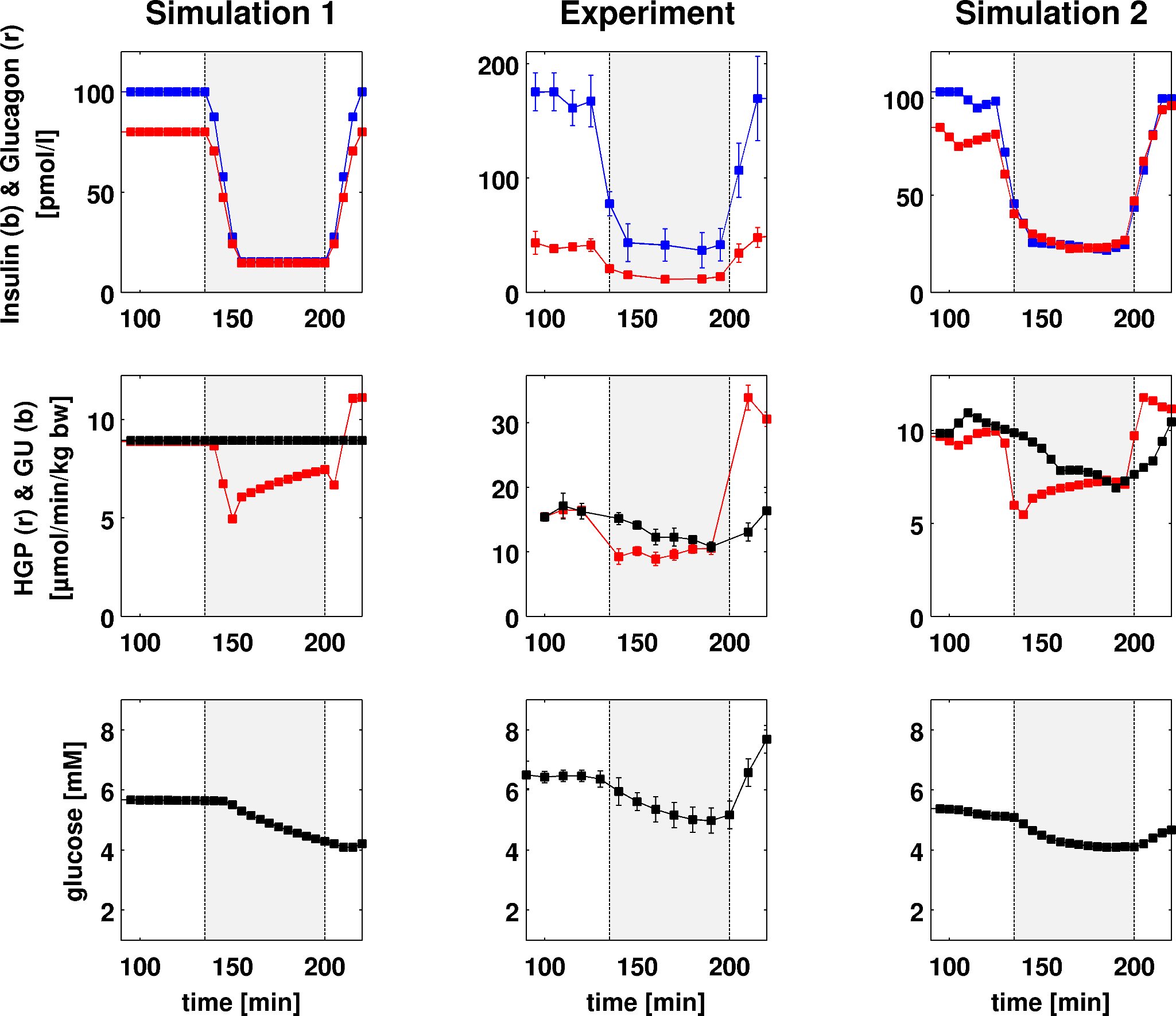

Supplement: Figure S2 — Cherrington1976 Insulin and glucagon deficiency. Figure analog to Figure 4C. (top row) Insulin (blue) and glucagon (red) time courses for an idealized hormone perturbation (left), the experimental data [27] (center), and taking the experimental profile of hormones and glucose utilization into account (right). (middle row) Time course of HGP (red) and whole-body glucose utilization GU (black) for the respective hormone profiles. (bottom row) Resulting changes in plasma glucose as a consequence of the changes in HGP for the respective hormone profiles and HGP/HGU time courses. Experimental data is provided in Dataset S1. (TIF) [file pcbi.1002577.s004.tif]

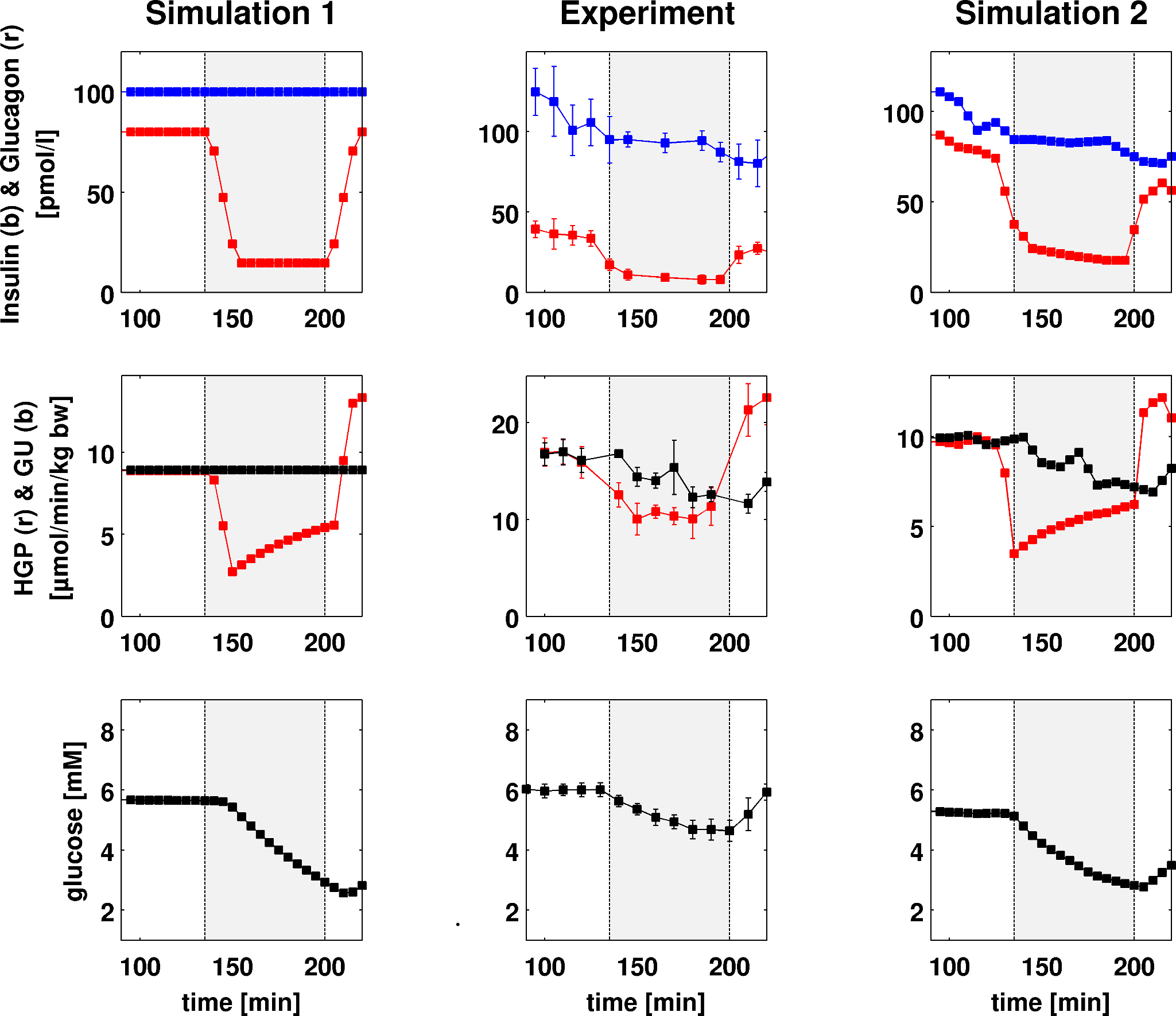

Supplement: Figure S3 — Cherrington1976 Glucagon deficiency. Figure analog to Figure 4C. (top row) Insulin (blue) and glucagon (red) time courses for an idealized hormone perturbation (left), the experimental data [27] (center), and taking the experimental profile of hormones and glucose utilization into account (right). (middle row) Time course of HGP (red) and whole-body glucose utilization GU (black) for the respective hormone profiles. (bottom row) Resulting changes in plasma glucose as a consequence of the changes in HGP for the respective hormone profiles and HGP/HGU time courses. Experimental data is provided in Dataset S1. (TIF) [file pcbi.1002577.s005.tif]

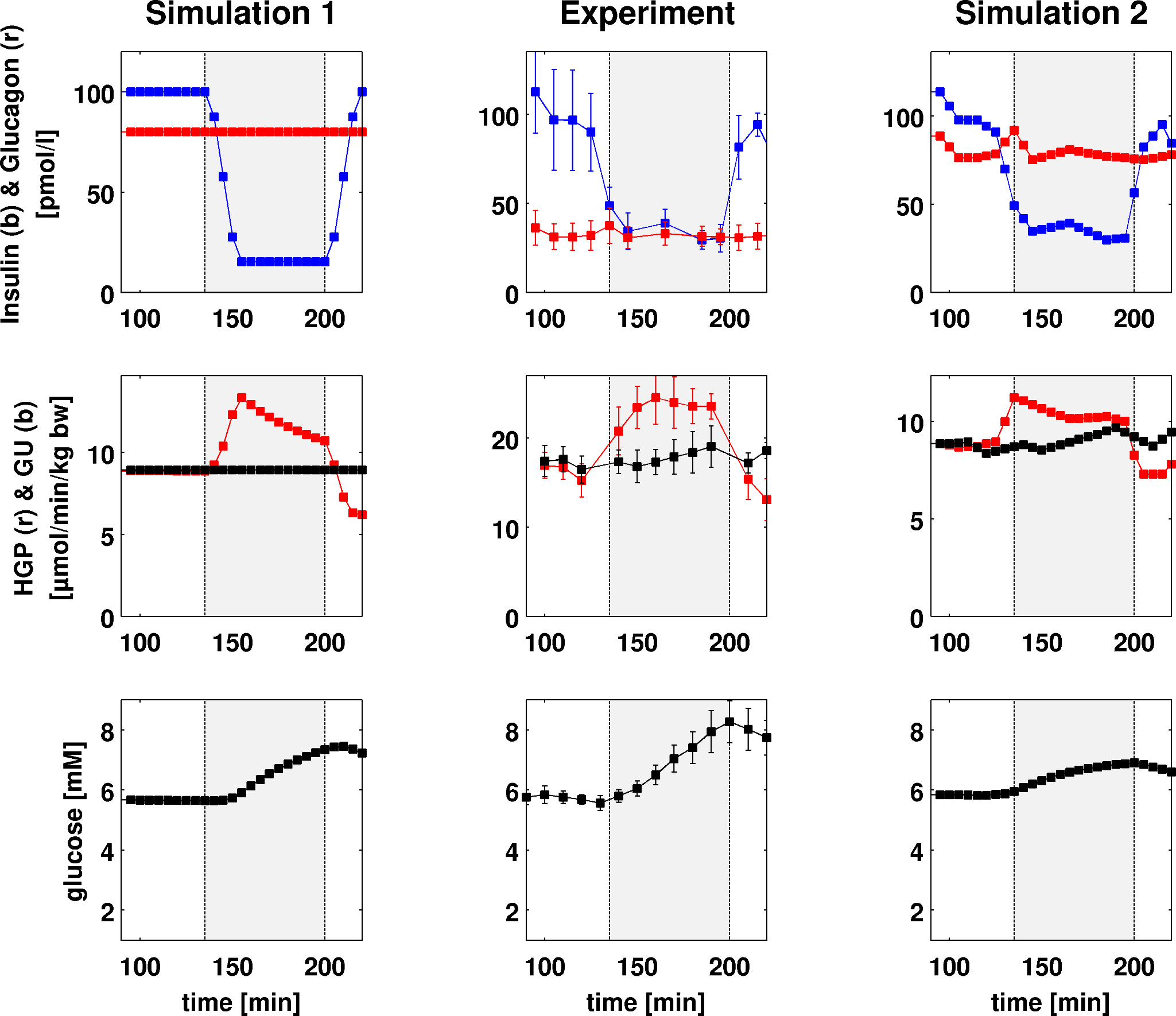

Supplement: Figure S4 — Cherrington1976 Insulin deficiency. Figure analog to Figure 4C. (top row) Insulin (blue) and glucagon (red) time courses for an idealized hormone perturbation (left), the experimental data [27] (center), and taking the experimental profile of hormones and glucose utilization into account (right). (middle row) Time course of HGP (red) and whole-body glucose utilization GU (black) for the respective hormone profiles. (bottom row) Resulting changes in plasma glucose as a consequence of the changes in HGP for the respective hormone profiles and HGP/HGU time courses. Experimental data is provided in Dataset S1. (TIF) [file pcbi.1002577.s006.tif]

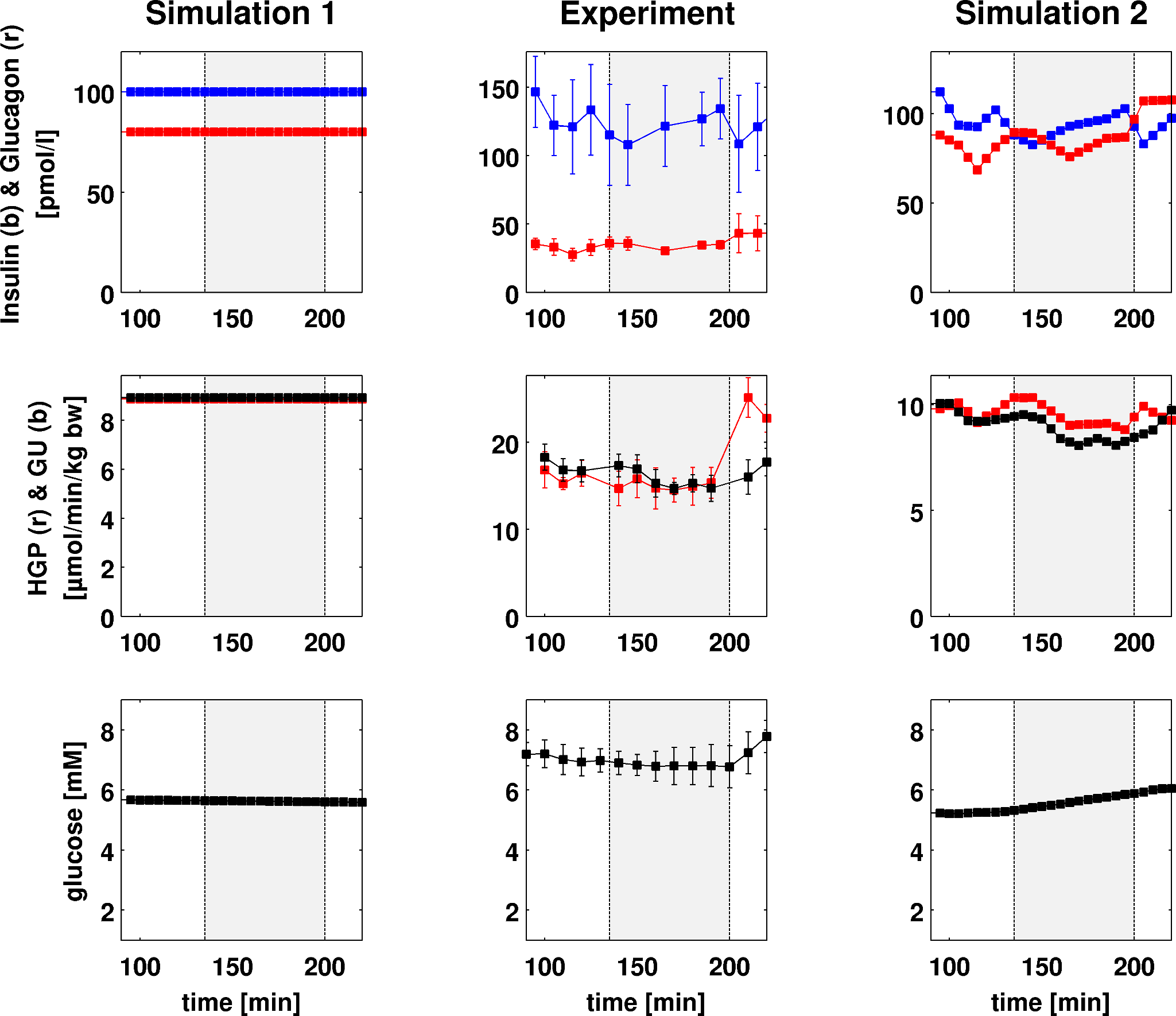

Supplement: Figure S5 — Cherrington1976 Somatostatin and insulin and glucagon restoration. Figure analog to Figure 4C. (top row) Insulin (blue) and glucagon (red) time courses for an idealized hormone perturbation (left), the experimental data [27] (center), and taking the experimental profile of hormones and glucose utilization into account (right). (middle row) Time course of HGP (red) and whole-body glucose utilization GU (black) for the respective hormone profiles. (bottom row) Resulting changes in plasma glucose as a consequence of the changes in HGP for the respective hormone profiles and HGP/HGU time courses. Experimental data is provided in Dataset S1. (TIF) [file pcbi.1002577.s007.tif]

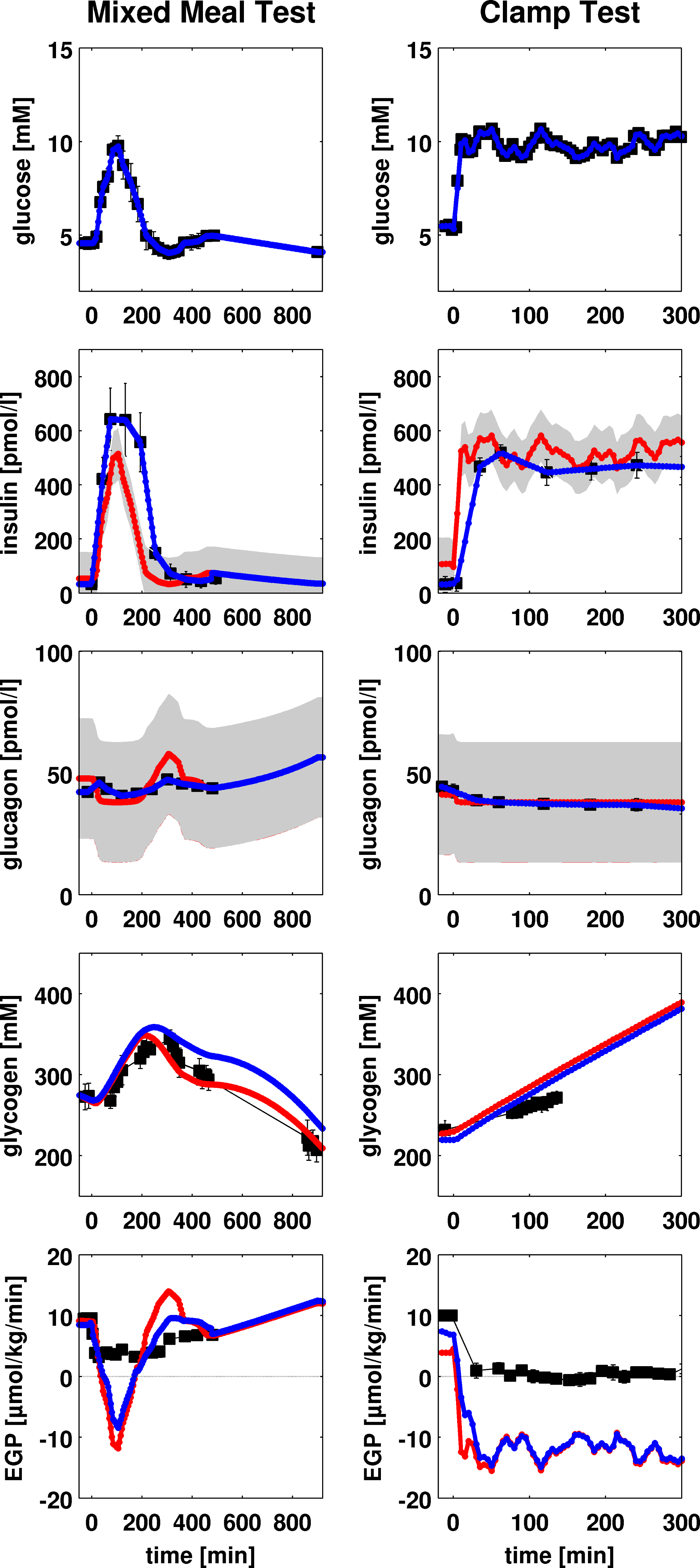

Supplement: Figure S6 — Krssak2004 Postprandial hepatic glycogen metabolism. Time courses of insulin, glucagon, glycogen and endogenous glucose production/net HGP for mixed meal tests (left) and hyperglycemic, hyperinsulinemic clamp tests (right) in Humans. Experimental data from [26] shown as black box with error bars (experimental data extracted from figures and tables). (I) Blue curves are model simulations with experimental glucose, insulin and glucagon time courses as model input without using hormone dose response curves. (II) Red curves model predictions under given glucose time course with predicted insulin and glucagon time courses via the respective hormone dose response curves as model input. In case of identical simulation results for (I) and (II) only the blue curves are shown. Initial glycogen in the mixed meal simulations are set to 275 mM glycogen and 220 mM glycogen in the clamp simulation. Grey error estimations are based on model simulations taking the errors in insulin and glucagon into account. Predicted insulin, glucagon and glycogen time courses are in agreement with the experimentally observed time courses. Predicted time courses of glycogen and EGP are very similar for (I) and (II). The glycogen synthesis rate in the clamp experiment is overestimated by the model. Imperfect simulation of glycogen synthesis in the mixed-meal and clamp simulations may be due to the fact that throughout the model the phosphorylation state of all regulatory enzymes was the same at given concentration of glucagon and insulin. This assumption is a simplification which could be the reason why the activation of the glycogen synthase was overestimated in our simulations. The measured endogenous glucose production (EGP) and the net hepatic glucose production (HGP) are substantially different. The presented model is a model describing the net hepatic output of the liver, not taking into account possible simultaneous production and utilization of glucose within the liver, for instance in perip [file pcbi.1002577.s008.tif]

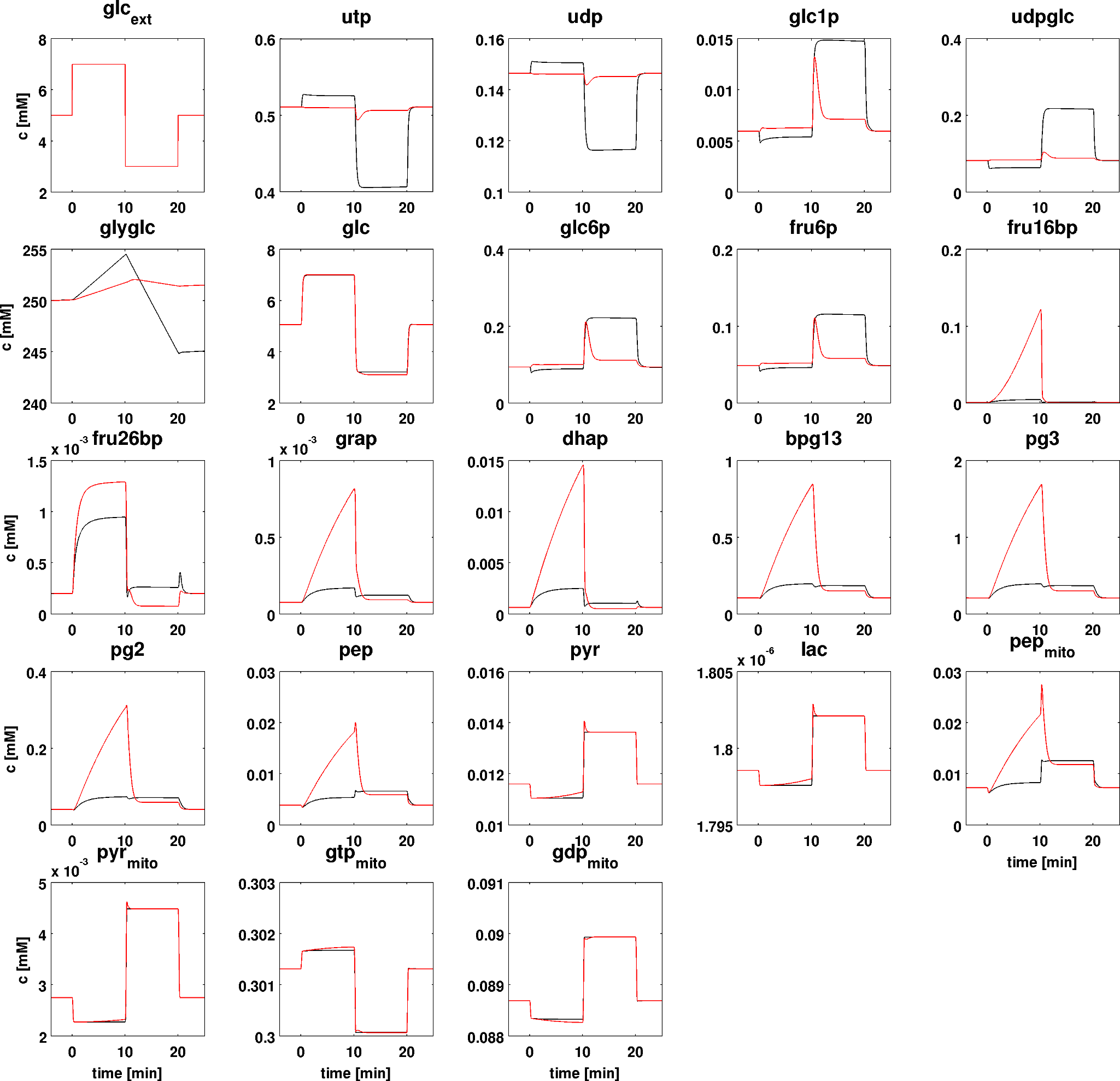

Supplement: Figure S7 — Fast time course simulation: model concentrations. Time course of model metabolites during perturbations in plasma glucose from initial 5 mM to 7 mM at t = 0 min, from 7 mM to 3 mM glucose at t = 10 min and from 3 mM back to the initial 5 mM glucose at t = 20 min. Respective fluxes are depicted in Figure S8. Normal model simulations are shown in black. After perturbation the model reaches the new quasi-steady state (only slow change in glycogen) within minutes, even after strong perturbations in blood glucose. To analyze the effect of the hormonal regulation on the glycogen metabolism and to distinguish the effects due to fast allosteric regulations from the hormonal regulation, the model was simulated without changing the phosphorylation state of the glycogen synthase (GS) and glycogen phosphorylase (GP) during the glucose perturbations (red model simulations). The reduced glycogen metabolism leads to an accumulation of the glycolytic intermediates during hyperglycemia due to an inability of the system to store the glucose. If both simulations yield equal results only the red curve is visible. (TIF) [file pcbi.1002577.s009.tif]

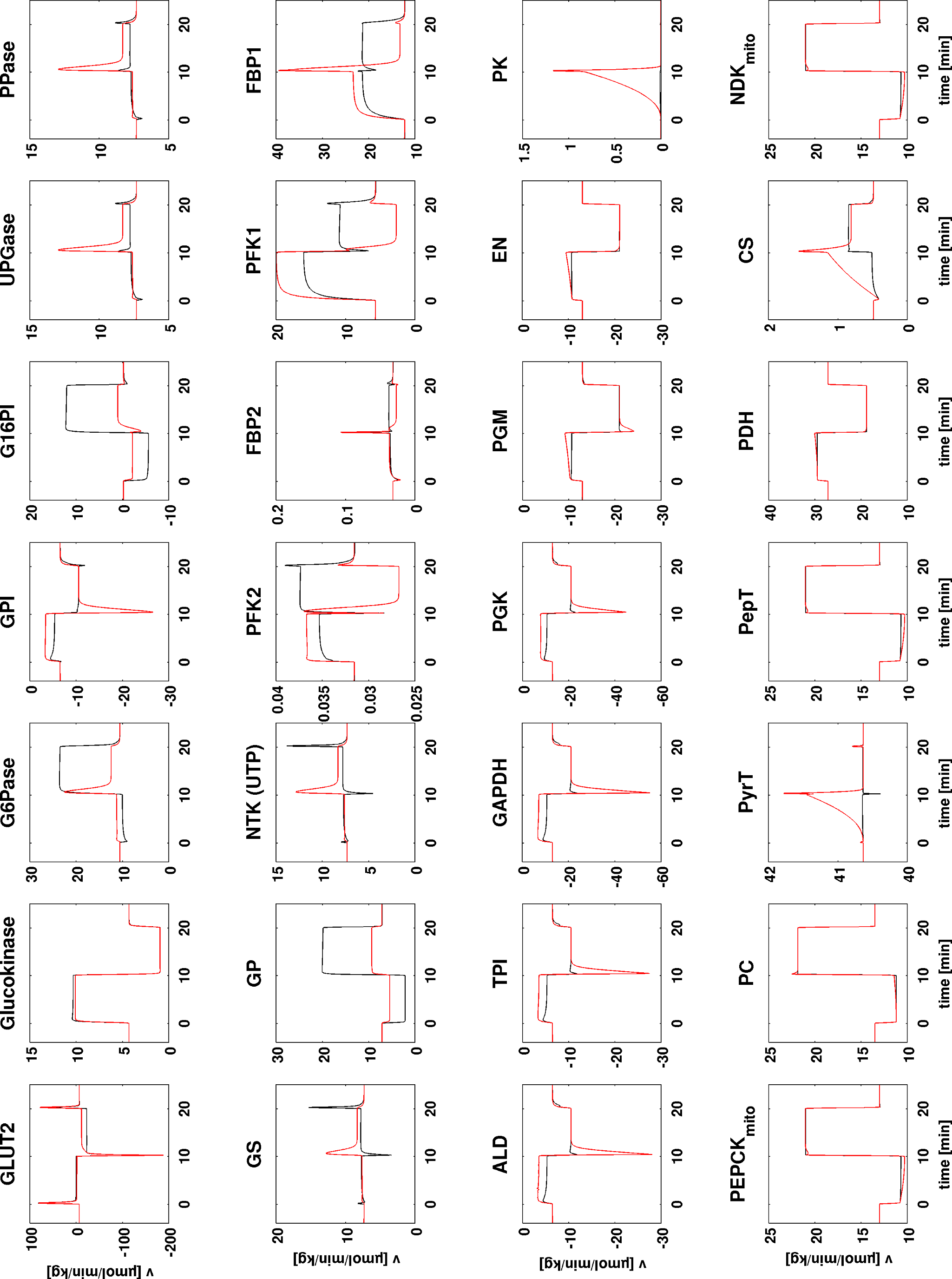

Supplement: Figure S8 — Fast time course simulation: model fluxes. Time course of model fluxes during glucose disturbance profiles. The description of the glucose profile and the corresponding concentration time courses are given in Figure S7. Even without hormonal regulation the glycogen system is able to switch direction between glycogen synthesis and glycogenolysis under hypoglycemia as a consequence of the futile cycle in glycogen metabolism. Hormonal regulation supports the inherent switching of directions increasing the flux in the respective directions. GP decreased in hypoglycemia and strongly increased in hypoglycemia. (TIF) [file pcbi.1002577.s010.tif]

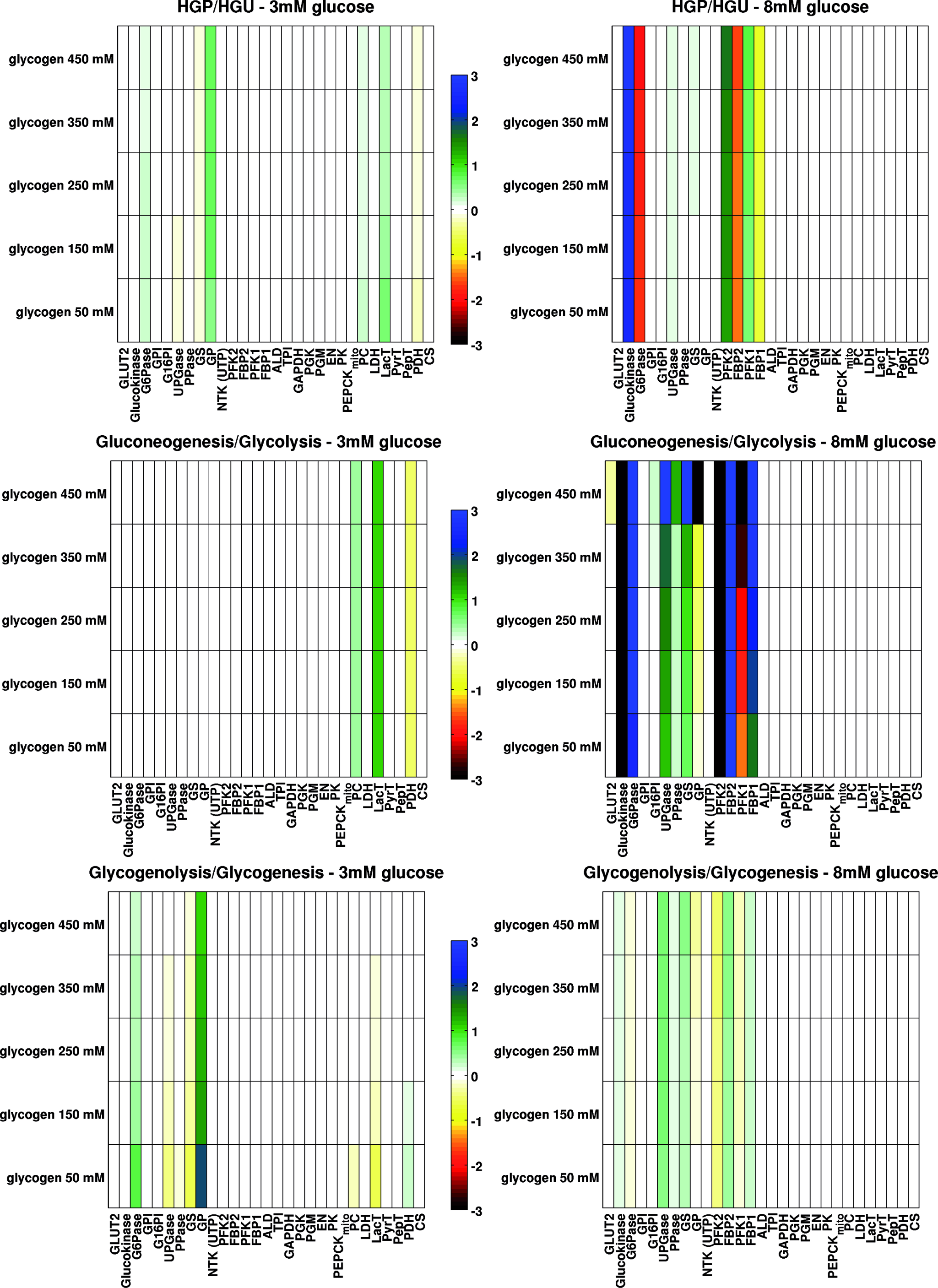

Supplement: Figure S9 — Metabolic control analysis of HGP. Metabolic response coefficients of the HGP/HGU flux (top), gluconeogenesis/glycolysis flux (middle) and glycogenolysis/glycogenesis flux (bottom) for hypoglycemic conditions of 3 mM (left) and hyperglycemic conditions of 8 mM (right) for various glycogen concentrations. Analyzed were the effects of changes in Vmax values of the enzymes of hepatic glucose metabolism on the steady state fluxes under the given conditions. The main interesting effects are: (i) The key enzymes depend strongly on the glucose concentration, different enzymes change the HGP/HGU, gluconeogenesis/glycolysis and glycogen synthesis/glycogenolysis under hypoglycemia and hyperglycemia. (ii) Different Vmax show the main response on HGP/HGU, gluconeogenesis/glycolysis and the glycogen metabolism (iii) The interconvertible enzymes show strong responses to changes in the Vmax values (iv) Only a selected number of enzymes has a strong response, the change in Vmax of many enzymes results in almost no change in key glucose fluxes. (TIF) [file pcbi.1002577.s011.tif]
