# Supplementary material for: Quantifying the Contribution of the Liver to Glucose Homeostasis: A Detailed Kinetic Model of Human Hepatic Glucose Metabolism
Source: PLoS Comput Biol. 2012 Jun 21;8(6):e1002577. doi: 10.1371/journal.pcbi.1002577 (PMC3383054; doi:10.1371/journal.pcbi.1002577)
Supplement: Text S1 — Overview metabolites. The full metabolite name, the used short name in the model, the and the respective compartment are depicted. The Initial concentrations with the respective references used for all simulations are either from human or rat liver/hepatocytes. The concentrations are given in more detailed table format in Dataset S1. In the case that no compartment-specific data for mitochondrion was available, the respective metabolite concentrations for cytosol were used. The glycogen concentration is given in glucose equivalents. (PDF) [file pcbi.1002577.s012.pdf]

Text S1. Overview metabolites.

Full Name	Short Name	Compart-ment	Constant	Concen-tration [mM]	References
ATP	atp	cyto	Y	2.8	[1-6]
ADP	adp	cyto	Y	0.8	[1,3,4,6,7]
AMP	amp	cyto	Y	0.16	[3,4,6]
UTP	utp	cyto		0.27	[4,7]
UDP	udp	cyto		0.09	[4,7]
GTP	gtp	cyto		0.29	[3,7]
GDP	gdp	cyto		0.10	[3,7]
NAD^+	nad	cyto	Y	1.22	[1,8]
NADH	nadh	cyto	Y	$0.56*10^{-3}$	[1,8]
phosphate	p	cyto	Y	5	[1,8]
pyrophosphate	pp	cyto		0.008	[9,10]
CO_2	co2	cyto	Y	5	[11]
glucose-1-phosphate	glc1p	cyto		0.012	[5,9,10]
UDP-glucose	udpglc	cyto		0.38	[5,9,10]
glycogen	glyglc	cyto		220	[12]
glucose	glc	cyto		5	[2,10,13]
glucose-6-phosphate	glc6p	cyto		0.12	[1,2,5,6,9]
fructose-6-phosphate	fru6p	cyto		0.05	[2,5,10]
fructose-1,6-bisphospate	fru16bp	cyto		0.02	[2,5,10]
fructose-2,6-bisphospate	fru26bp	cyto		0.004	[6]
glyceraldehyde 3-phosphate	grap	cyto		0.1	[1,5]
dihydroxy-acetone phosphate	dhap	cyto		0.03	[1,2,5,10]
1,3-bisphospho-glycerate	bpg13	cyto		0.3	
3-phosphoglycerate	pg3	cyto		0.27	[1,10]
2-phosphoglycerate	pg2	cyto		0.03	[1,10]
phosphoenol pyruvate	pep	cyto		0.15	[1,5,6]
pyruvate	pyr	cyto		0.1	[1,2,5,6,10]
oxalacetate	oaa	cyto		0.01	[1,2,5,10]
lactate	lac	cyto		0.5	[1,2,5,6]
glucose	glc_ext	blood		5	[13]
lactate	lac_ext	blood		1.2	[8,14]
CO_2	co2_mito	mito	Y	5	[11]
phosphate	p_mito	mito	Y	5	[1,8]
oxalacetate	oaa_mito	mito		0.01	[1,2,5,6,10]
pep	pep_mito	mito		0.15	[1,5,6]
acetyl-coenzyme A	acoa_mito	mito	Y	0.04	[1,2,5]
pyruvate	pyr_mito	mito		0.1	[1,2,5,6,10]
citrate	cit_mito	mito	Y	0.32	[1,2,5,6]
ATP	atp_mito	mito	Y	2.8	[1-6]
ADP	adp_mito	mito	Y	0.8	[1,3,4,6,7]
GTP	gtp_mito	mito		0.29	[3,7]
GDP	gdp_mito	mito		0.10	[3,7]
coenzyme A	coa_mito	mito	Y	0.055	[1]
NADH	nadh_mito	mito	Y	0.24	[10,14]
NAD^+	nad_mito	mito	Y	0.98	[8,10]

References

1. Veech RL, Veloso D, Mehlman MA (1973) Thiamin deficiency: liver metabolite levels and redox and phosphorylation states in thiamin-deficient rats. J Nutr 103: 267-272.
2. Rawat AK (1968) Effects of ethanol infusion on the redox state and metabolite levels in rat liver in vivo. Eur J Biochem 6: 585-592.
3. Bartel T, Holzhutter HG (1990) Mathematical modelling of the purine metabolism of the rat liver. Biochim Biophys Acta 1035: 331-339.
4. Eriksson G (1980) A method for measuring nucleotides in the liver of rat with isotachophoresis. Anal Biochem 109: 239-246.
5. Faupel RP, Seitz HJ, Tarnowski W, Thiemann V, Weiss C (1972) The problem of tissue sampling from experimental animals with respect to freezing technique, anoxia, stress and narcosis. A new method for sampling rat liver tissue and the physiological values of glycolytic intermediates and related compounds. Arch Biochem Biophys 148: 509-522.
6. Hue L (1982) Role of fructose 2,6-bisphosphate in the stimulation of glycolysis by anoxia in isolated hepatocytes. Biochem J 206: 359-365.
7. Jackson RC, Morris HP, Weber G (1977) Partial purification, properties and regulation of inosine 5'phosphate dehydrogenase in normal and malignant rat tissues. Biochem J 166: 1-10.
8. Chalhoub E, Hanson RW, Belovich JM (2007) A computer model of gluconeogenesis and lipid metabolism in the perfused liver. Am J Physiol Endocrinol Metab 293: E1676-1686.
9. Guynn RW, Veloso D, Lawson JW, Veech RL (1974) The concentration and control of cytoplasmic free inorganic pyrophosphate in rat liver in vivo. Biochem J 140: 369-375.
10. Albe KR, Butler MH, Wright BE (1990) Cellular concentrations of enzymes and their substrates. J Theor Biol 143: 163-195.
11. Schmidt RF, Lang F, Thews G (2007) Physiologie des Menschen mit Pathophysiologie ; mit 77 Tabellen ; [+ IMPP-Fragen Physiologie online]. 30., neu bearb. und aktualisierte Aufl.. ed. Heidelberg: Springer-Medizin-Verl. pp. XXII, 1030 S.
12. Rothman DL, Magnusson I, Katz LD, Shulman RG, Shulman GI (1991) Quantitation of hepatic glycogenolysis and gluconeogenesis in fasting humans with 13C NMR. Science 254: 573-576.
13. Gerich JE (1993) Control of glycaemia. Baillieres Clin Endocrinol Metab 7: 551-586.
14. Chalhoub E, Xie L, Balasubramanian V, Kim J, Belovich J (2007) A distributed model of carbohydrate transport and metabolism in the liver during rest and high-intensity exercise. Ann Biomed Eng 35: 474-491.
