# Supplementary material for: Quantifying the Contribution of the Liver to Glucose Homeostasis: A Detailed Kinetic Model of Human Hepatic Glucose Metabolism
Source: PLoS Comput Biol. 2012 Jun 21;8(6):e1002577. doi: 10.1371/journal.pcbi.1002577 (PMC3383054; doi:10.1371/journal.pcbi.1002577)
Supplement: Text S2 — Overview reactions and transporters. The full reaction/transporter name, the used short name in the model, the and the respective compartment are depicted. The rate equations of the kinetic model are given in Text S3. The given references are the references for the kinetic parameters. Furthermore, the fitted Vmax values used for simulations are given. (PDF) [file pcbi.1002577.s013.pdf]

**Text S2. Overview reactions and transporters.**

| Full Name | Short Name | | Compartment | $V_{max}$ [µmol/min/kg] | References |
|---|---|---|---|---|---|
| GLUT2 glucose transporter | GLUT2 | eq.4 | pm | 250 | [1-5] |
| Glucokinase | GK | eq.5 | cyto | 15 | [4,6-11] |
| Glucose-6-phosphatase | G6PASE | eq.6 | cyto | 11.25 | [12-14] |
| Glucose-6-phosphate isomerase | GPI | eq.7 | cyto | 250 | [15,16] |
| Glucose-1-phosphate 1,6-phosphomutase | G16PI | eq.8 | cyto | 200 | [17] |
| UTP:Glucose-1-phosphate uridylyltransferase | UGT | eq.9 | cyto | 160 | [18-20] |
| Pyrophosphate phosphohydrolase | PPASE | eq.10 | cyto | 4.8 | [21,22] |
| Glycogen synthase | GS | eq.11 | cyto | 26.4 | [23-27] |
| Glycogen phosphorylase | GP | eq.12 | cyto | 13.6 | [28-33] |
| Nucleosid diphosphate kinase (GTP) | NDK | eq.13 | cyto | 1750 | [34-36] |
| Nucleosid diphosphate kinase (UTP) | NDK | eq.14 | cyto | 1750 | [34-36] |
| Adenylat kinase | AK | eq.15 | cyto | 500 | [16,37,38] |
| Phosphofructo kinase 2 | PFK2 | eq.16 | cyto | 0.0025 | [39-43] |
| Fructose-2,6-bisphosphatase | FBP2 | eq.17 | cyto | 0.075 | [39-43] |
| Phosphofructo kinase 1 | PFK1 | eq.18 | cyto | 0.525 | [44-46] |
| Fructose-1,6-bisphosphatase | FBP1 | eq.19 | cyto | 2.575 | [47,48] |
| Aldolase | ALD | eq.20 | cyto | 250 | [16,49-51] |
| Triosephosphate isomerase | TPI | eq.21 | cyto | 250 | [16,52,53] |
| Glyceraldehydephosphate dehydrogenase | GAPDH | eq.22 | cyto | 250 | [16] |
| Phosphoglycerate kinase | PGK | eq.23 | cyto | 250 | [16] |
| 3-Phosphoglycerate mutase | PGM | eq.24 | cyto | 250 | [16] |
| Enolase | EN | eq.25 | cyto | 21.43 | [16] |
| Pyruvate kinase | PK | eq.26 | cyto | 20 | [54-57] |
| Phosphoenolpyruvate carboxykinase | PEPCK | eq.28 | cyto | 325 | [58,59] |
| Phosphoenolpyruvate carboxykinase | PEPCK | eq.28 | mito | 325 | [58,59] |
| Pyruvate Carboxylase | PC | eq.29 | mito | 100 | [60,61] |
| Lactate dehydrogenase | LDH | eq.30 | cyto | 7.5 | [62-64] |
| Lactate transporter | LACT | eq.31 | pm | 33 | |
| Pyruvate transporter | PYRT | eq.32 | mm | 25 | |
| PEP transporter | PEPT | eq.33 | mm | 20 | |
| Pyruvate dehydrogenase | PDH | eq.34 | mito | 2.5 | [65-68] |
| Citrate synthase | CS | eq.35 | mito | 2.5 | [4,69-71] |
| Nucleosid diphosphate kinase (GTP) | NDK | eq.36 | mito | 250 | [34-36] |
| Oxalacetate flux | OAAFLX | eq.37 | mito | 0 | |
| Acetyl-CoA flux | ACOAFLX | eq.38 | mito | 0 | |
| Citrate flux | CITFLX | eq.39 | mito | 0 | |

## References


1. Ciaraldi TP, Horuk R, Matthaei S (1986) Biochemical and functional characterization of the rat liver glucose-transport system. Comparisons with the adipocyte glucose-transport system. Biochem J 240: 115-123.
2. Gould GW, Thomas HM, Jess TJ, Bell GI (1991) Expression of human glucose transporters in Xenopus oocytes: kinetic characterization and substrate specificities of the erythrocyte, liver, and brain isoforms. Biochemistry 30: 5139-5145.
3. Leturque A, Brot-Laroche E, Le Gall M (2009) GLUT2 mutations, translocation, and receptor function in diet sugar managing. Am J Physiol Endocrinol Metab 296: E985-992.
4. Nelson DL, Cox MM, Lehninger AL (2008) Principles of biochemistry. New York: Freeman. getr. Zählung. p.
5. Thorens B (1996) Glucose transporters in the regulation of intestinal, renal, and liver glucose fluxes. Am J Physiol 270: G541-553.
6. Agius L (2008) Glucokinase and molecular aspects of liver glycogen metabolism. Biochem J 414: 1-18.
7. Agius L, Peak M (1993) Intracellular binding of glucokinase in hepatocytes and translocation by glucose, fructose and insulin. Biochem J 296 ( Pt 3): 785-796.
8. Brocklehurst KJ, Davies RA, Agius L (2004) Differences in regulatory properties between human and rat glucokinase regulatory protein. Biochem J 378: 693-697.
9. Morita H, Yano Y, Niswender KD, May JM, Whitesell RR, et al. (1994) Coexpression of glucose transporters and glucokinase in Xenopus oocytes indicates that both glucose transport and phosphorylation determine glucose utilization. J Clin Invest 94: 1373-1382.
10. Storer AC, Cornish-Bowden A (1976) Kinetics of rat liver glucokinase. Co-operative interactions with glucose at physiologically significant concentrations. Biochem J 159: 7-14.
11. Van Schaftingen E (1989) A protein from rat liver confers to glucokinase the property of being antagonistically regulated by fructose 6-phosphate and fructose 1-phosphate. Eur J Biochem 179: 179-184.
12. Arion WJ, Wallin BK, Carlson PW, Lange AJ (1972) The specificity of glucose 6-phosphatase of intact liver microsomes. J Biol Chem 247: 2558-2565.
13. Nordlie RC (1969) Some properties and possible physiological functions of phosphotransferase activities of microsomal glucose-6-phosphatase. Ann N Y Acad Sci 166: 699-718.
14. Reczek PR, Villee CA, Jr. (1982) A purification of microsomal glucose-6-phosphatase from human tissue. Biochem Biophys Res Commun 107: 1158-1165.
15. Kahana SE, Lowry OH, Schulz DW, Passonneau JV, Crawford EJ (1960) The kinetics of phosphoglucoisomerase. J Biol Chem 235: 2178-2184.
16. Schuster R, Holzhutter HG (1995) Use of mathematical models for predicting the metabolic effect of large-scale enzyme activity alterations. Application to enzyme deficiencies of red blood cells. Eur J Biochem 229: 403-418.
17. Kashiwaya Y, Sato K, Tsuchiya N, Thomas S, Fell DA, et al. (1994) Control of glucose utilization in working perfused rat heart. J Biol Chem 269: 25502-25514.

18. Chang HY, Peng HL, Chao YC, Duggleby RG (1996) The importance of conserved residues in human liver UDPglucose pyrophosphorylase. Eur J Biochem 236: 723-728.
19. Duggleby RG, Chao YC, Huang JG, Peng HL, Chang HY (1996) Sequence differences between human muscle and liver cDNAs for UDPglucose pyrophosphorylase and kinetic properties of the recombinant enzymes expressed in Escherichia coli. Eur J Biochem 235: 173-179.
20. Turnquist RL, Gillett TA, Hansen RG (1974) Uridine diphosphate glucose pyrophosphorylase. Crystallization and properties of the enzyme from rabbit liver and species comparisons. J Biol Chem 249: 7695-7700.
21. Reichert WH, Lauter CJ, Trams EG (1974) Inorganic pyrophosphatase in cultured cells. Biochim Biophys Acta 370: 556-563.
22. Tamura T, Shiraki H, Nakagawa H (1980) Purification and characterization of adenylate kinase isozymes from rat muscle and liver. Biochim Biophys Acta 612: 56-66.
23. Bosch F, Ciudad CJ, Guinovart JJ (1983) Different effects of glucagon and epinephrine on the kinetic properties of liver glycogen synthase. FEBS Lett 151: 76-78.
24. Ferrer JC, Favre C, Gomis RR, Fernandez-Novell JM, Garcia-Rocha M, et al. (2003) Control of glycogen deposition. FEBS Lett 546: 127-132.
25. Rawat AK (1968) Effects of ethanol infusion on the redox state and metabolite levels in rat liver in vivo. Eur J Biochem 6: 585-592.
26. Rothman DL, Magnusson I, Katz LD, Shulman RG, Shulman GI (1991) Quantitation of hepatic glycogenolysis and gluconeogenesis in fasting humans with 13C NMR. Science 254: 573-576.
27. Westphal SA, Nuttall FQ (1992) Comparative characterization of human and rat liver glycogen synthase. Arch Biochem Biophys 292: 479-486.
28. Ercan-Fang N, Gannon MC, Rath VL, Treadway JL, Taylor MR, et al. (2002) Integrated effects of multiple modulators on human liver glycogen phosphorylase a. Am J Physiol Endocrinol Metab 283: E29-37.
29. Lederer B, Stalmans W (1976) Human liver glycogen phosphorylase. Kinetic properties and assay in biopsy specimens. Biochem J 159: 689-695.
30. Maddaiah VT, Madsen NB (1966) Kinetics of purified liver phosphorylase. J Biol Chem 241: 3873-3881.
31. Stalmans W, Gevers G (1981) The catalytic activity of phosphorylase b in the liver. With a note on the assay in the glycogenolytic direction. Biochem J 200: 327-336.
32. Stalmans W, Hers HG (1975) The stimulation of liver phosphorylase b by AMP, fluoride and sulfate. A technical note on the specific determination of the a and b forms of liver glycogen phosphorylase. Eur J Biochem 54: 341-350.
33. Tan AW, Nuttall FQ (1975) Characteristics of the dephosphorylated form of phosphorylase purified from rat liver and measurement of its activity in crude liver preparations. Biochim Biophys Acta 410: 45-60.
34. Fukuchi T, Shimada N, Hanai N, Ishikawa N, Watanabe K, et al. (1994) Recombinant rat nucleoside diphosphate kinase isoforms (alpha and beta): purification, properties and application to immunological detection of native isoforms in rat tissues. Biochim Biophys Acta 1205: 113-122.

35. Kimura N, Shimada N (1988) Membrane-associated nucleoside diphosphate kinase from rat liver. Purification, characterization, and comparison with cytosolic enzyme. J Biol Chem 263: 4647-4653.
36. Lam SC, Packham MA (1986) Isolation and kinetic studies of nucleoside diphosphokinase from human platelets and effects of cAMP phosphodiesterase inhibitors. Biochem Pharmacol 35: 4449-4455.
37. Blair JM (1970) Magnesium, potassium, and the adenylate kinase equilibrium. Magnesium as a feedback signal from the adenine nucleotide pool. Eur J Biochem 13: 384-390.
38. Tsuboi KK, Chervenka CH (1975) Adenylate kinase of human erythrocyte. Isolation and properties of the predominant inherited form. J Biol Chem 250: 132-140.
39. Richards CS, Furuya E, Uyeda K (1981) Regulation of fructose 2,6-P2 concentration in isolated hepatocytes. Biochem Biophys Res Commun 100: 1673-1679.
40. Richards CS, Yokoyama M, Furuya E, Uyeda K (1982) Reciprocal changes in fructose-6-phosphate,2-kinase and fructose-2,6-bisphosphatase activity in response to glucagon and epinephrine. Biochem Biophys Res Commun 104: 1073-1079.
41. Sakakibara R, Kitajima S, Uyeda K (1984) Differences in kinetic properties of phospho and dephospho forms of fructose-6-phosphate, 2-kinase and fructose 2,6-bisphosphatase. J Biol Chem 259: 41-46.
42. Sakata J, Abe Y, Uyeda K (1991) Molecular cloning of the DNA and expression and characterization of rat testes fructose-6-phosphate,2-kinase:fructose-2,6-bisphosphatase. J Biol Chem 266: 15764-15770.
43. Van Schaftingen E, Jett MF, Hue L, Hers HG (1981) Control of liver 6-phosphofructokinase by fructose 2,6-bisphosphate and other effectors. Proc Natl Acad Sci U S A 78: 3483-3486.
44. Brand IA, Soling HD (1974) Rat liver phosphofructokinase. Purification and characterization of its reaction mechanism. J Biol Chem 249: 7824-7831.
45. Dunaway GA, Segal HL (1976) Purification and physiological role of a peptide stabilizing factor of rat liver phosphofructokinase. J Biol Chem 251: 2323-2329.
46. Durante P, Raleigh X, Gomez ME, Campos G, Ryder E (1995) Isozyme analysis of human normal polymorphonuclear leukocyte phosphofructokinase. Biochem Biophys Res Commun 216: 898-905.
47. Adams A, Redden C, Menahem S (1990) Characterization of human fructose-1,6-bisphosphatase in control and deficient tissues. J Inherit Metab Dis 13: 829-848.
48. Dzugaj A, Kochman M (1980) Purification of human liver fructose-1,6-bisphosphatase. Biochim Biophys Acta 614: 407-412.
49. Bais R, James HM, Rofe AM, Conyers RA (1985) The purification and properties of human liver ketohexokinase. A role for ketohexokinase and fructose-bisphosphate aldolase in the metabolic production of oxalate from xylitol. Biochem J 230: 53-60.
50. Doyle SA, Tolan DR (1995) Characterization of recombinant human aldolase B and purification by metal chelate chromatography. Biochem Biophys Res Commun 206: 902-908.
51. Malay AD, Procious SL, Tolan DR (2002) The temperature dependence of activity and structure for the most prevalent mutant aldolase B associated with hereditary

fructose intolerance. Arch Biochem Biophys 408: 295-304.
52. Snyder R, Lee EW (1975) Triosephosphate isomerase from human and horse liver. Methods Enzymol 41: 430-434.
53. Yuan PM, Dewan RN, Zaun M, Thompson RE, Gracy RW (1979) Isolation and characterization of triosephosphate isomerase isozymes from human placenta. Arch Biochem Biophys 198: 42-52.
54. Flory W, Peczon BD, Koeppe RE, Spivey HO (1974) Kinetic properties of rat liver pyruvate kinase at cellular concentrations of enzyme, substrates and modifiers. Biochem J 141: 127-131.
55. Ishibashi H, Cottam GL (1978) Glucagon-stimulated phosphorylation of pyruvate kinase in hepatocytes. J Biol Chem 253: 8767-8771.
56. Reynard AM, Hass LF, Jacobsen DD, Boyer PD (1961) The correlation of reaction kinetics and substrate binding with the mechanism of pyruvate kinase. J Biol Chem 236: 2277-2283.
57. Van Berkel TJ, Kruijt JK, Van den Berg GB, Koster JF (1978) Difference in the effect of glucagon and starvation upon L-type pyruvate kinase from rat liver. Eur J Biochem 92: 553-561.
58. Case CL, Mukhopadhyay B (2007) Kinetic characterization of recombinant human cytosolic phosphoenolpyruvate carboxykinase with and without a His10-tag. Biochim Biophys Acta 1770: 1576-1584.
59. Yang J, Kalhan SC, Hanson RW (2009) What is the metabolic role of phosphoenolpyruvate carboxykinase? J Biol Chem 284: 27025-27029.
60. Jitrapakdee S, Walker ME, Wallace JC (1999) Functional expression, purification, and characterization of recombinant human pyruvate carboxylase. Biochem Biophys Res Commun 266: 512-517.
61. Scrutton MC, White MD (1974) Prufication and properties of human liver pyruvate carboxylase. Biochem Med 9: 217-292.
62. Ketchum CH, Robinson CA, Hall LM, Grizzle WE (1988) Lactate dehydrogenase isolated from human liver mitochondria: its purification and partial biochemical characterization. Clin Biochem 21: 231-237.
63. Pettit SM, Nealon DA, Henderson AR (1981) Purification of lactate dehydrogenase isoenzyme-5 from human liver. Clin Chem 27: 88-93.
64. Saad LO, Mirandola SR, Maciel EN, Castilho RF (2006) Lactate dehydrogenase activity is inhibited by methylmalonate in vitro. Neurochem Res 31: 541-548.
65. Hamada M, Koike K, Nakaula Y, Hiraoka T, Koike M (1975) A kinetic study of the alpha-keto acid dehydrogenase complexes from pig heart mitochondria. J Biochem 77: 1047-1056.
66. Holness MJ, MacLennan PA, Palmer TN, Sugden MC (1988) The disposition of carbohydrate between glycogenesis, lipogenesis and oxidation in liver during the starved-to-fed transition. Biochem J 252: 325-330.
67. Kiselevsky YV, Ostrovtsova SA, Strumilo SA (1990) Kinetic characterization of the pyruvate and oxoglutarate dehydrogenase complexes from human heart. Acta Biochim Pol 37: 135-139.
68. Korotchkina LG, Sidhu S, Patel MS (2006) Characterization of testis-specific isoenzyme of human pyruvate dehydrogenase. J Biol Chem 281: 9688-9696.
69. Matsuoka Y, Srere PA (1973) Kinetic studies of citrate synthase from rat kidney and

rat brain. J Biol Chem 248: 8022-8030.
70. Shepherd D, Garland PB (1969) The kinetic properties of citrate synthase from rat liver mitochondria. Biochem J 114: 597-610.
71. Smitherman TC, Mukherjee A, Robinson JB, Jr., Butsch RW, Richards EG, et al. (1979) Human heart citrate synthase: purification, properties, kinetic and immunologic studies. J Mol Cell Cardiol 11: 149-160.
