# Supplementary material for: Quantifying the Contribution of the Liver to Glucose Homeostasis: A Detailed Kinetic Model of Human Hepatic Glucose Metabolism
Source: PLoS Comput Biol. 2012 Jun 21;8(6):e1002577. doi: 10.1371/journal.pcbi.1002577 (PMC3383054; doi:10.1371/journal.pcbi.1002577)
Supplement: Text S3 — Kinetic rate equations of the model. Detailed rate equations of all processes of the model of human hepatic glucose metabolism. (PDF) [file pcbi.1002577.s014.pdf]

Text S3. Kinetic rate equations.

Glucose transporter v_1 (GLUT2)

$$glc\_ext \leftrightarrow glc$$

$$v_1([glc],[glc\_ext]) = \frac{\frac{v_{max}}{k_m^{glc}} \cdot \left([glc\_ext] - \frac{[glc]}{k_{eq}}\right)}{1 + \frac{[glc\_ext]}{k_m^{glc}} + \frac{[glc]}{k_m^{glc}}} \quad (4)$$

$$\triangle G^{0'} = 0\,\mathrm{kJ/mol}$$
$$k_{eq} = 1$$
$$k_m^{glc} = 42\,\mathrm{mM}$$

Glucokinase v_2 (Hexokinase IV, GK) Glucokinase activity is mainly regulated by GKRP (glucokinase regulator protein) which is a competitive inhibitor for glucose. The binding of GKRP depends on [glc], [fru6p] and [fru1p] and modifies the k_m for glucose. GKRP causes the translocation of GK into the nucleus and reduces the free GK concentration in the cytosol. Only free GK is active in the model.

$$glc + atp \rightarrow adp + glc6p$$

$$gk_{free}([glc],[fru6p]) = \frac{[glc]^{n_{gkrp}}}{[glc]^{n_{gkrp}} + (k_{gkrp}^{glc})^{n_{gkrp}}} \left(1 - \frac{b_{gkrp}[fru6p]}{[fru6p] + k_{gkrp}^{fru6p}})\right)$$

$$v_2([glc],[atp],[fru6p]) = v_{max} \cdot gk_{free} \cdot \frac{[atp]}{k_m^{atp} + [atp]} \cdot \frac{[glc]^n}{[glc]^n + (k_m^{glc})^n} \quad (5)$$

$$\triangle G^{0'} = -16.7\,\mathrm{kJ/mol}$$
$$n = 1.6$$
$$k_m^{glc} = 7.5\,\mathrm{mM}$$
$$k_m^{atp} = 0.26\,\mathrm{mM}$$
$$n_{gkrp} = 2$$
$$k_{gkrp}^{glc} = 15\,\mathrm{mM}$$
$$k_{gkrp}^{fru6p} = 0.010\,\mathrm{mM}$$
$$b_{gkrp} = 0.7$$

Glucose-6 phosphatase v_3 (G6PASE) G6Pase is modeled as a cytosolic enzyme. G6Pase is localized in the membrane of the endoplasmatic reticulum with the catalytic site facing towards the ER lumen (transporters for glc6p, p and glc exist in the ER membrane).

$$glc6p + h2o \rightarrow glc + p$$

$$v_3([glc6p]) = v_{max} \frac{[glc6p]}{k_m^{glc6p} + [glc6p]} \quad (6)$$

$$\triangle G^{0'} = -13.8\,\mathrm{kJ/mol}$$
$$k_m^{glc6p} = 2\,\mathrm{mM}$$

Glucose-6-phosphate isomerase v_4 (GPI)

$$glc6p \leftrightarrow fru6p$$

$$v_4([glc6p],[fru6p]) = \frac{\frac{v_{max}}{k_m^{glc6p}} \cdot \left([glc6p] - \frac{[fru6p]}{k_{eq}}\right)}{1 + \frac{[glc6p]}{k_m^{glc6p}} + \frac{[fru6p]}{k_m^{fru6p}}} \tag{7}$$

$$\triangle G^{0'} = 1.7\,\mathrm{kJ/mol}$$
$$k_{eq} = 0.5157$$
$$k_m^{glc6p} = 0.182\,\mathrm{mM}$$
$$k_m^{fru6p} = 0.071\,\mathrm{mM}$$

Glucose-1-phosphate 1,6-phosphomutase v_5 (G16PI)

$$glc1p \leftrightarrow glc6p$$

$$v_5([glc1p],[glc6p]) = \frac{\frac{v_{max}}{k_m^{glc1p}} \cdot \left([glc1p] - \frac{[glc6p]}{k_{eq}}\right)}{1 + \frac{[glc1p]}{k_m^{glc1p}} + \frac{[glc6p]}{k_m^{glc6p}}} \tag{8}$$

$$\triangle G^{0'} = -7.1\,\mathrm{kJ/mol}$$
$$k_{eq} = 15.7$$
$$k_m^{glc6p} = 0.67\,\mathrm{mM}$$
$$k_m^{glc1p} = 0.045\,\mathrm{mM}$$

UTP:Glucose-1-phosphate uridylyltransferase v_6 (UGT)

$$glc1p + utp \leftrightarrow udpglc + pp$$

$$v_6([glc1p],[utp],[udpglc],[pp]) = \frac{\frac{v_{max}}{k_m^{utp} k_m^{glc1p}} \left([glc1p][utp] - \frac{[udpglc][pp]}{k_{eq}}\right)}{\left(1 + \frac{[utp]}{k_m^{utp}}\right)\left(1 + \frac{[glc1p]}{k_m^{glc1p}}\right) + \left(1 + \frac{[udpglc]}{k_m^{udpglc}}\right)\left(1 + \frac{[pp]}{k_m^{pp}}\right) - 1} \tag{9}$$

$$\triangle G^{0'} = 3.0\,\mathrm{kJ/mol}$$
$$k_{eq} = 0.31$$
$$k_m^{utp} = 0.563\,\mathrm{mM}$$
$$k_m^{glc1p} = 0.172\,\mathrm{mM}$$
$$k_m^{udpglc} = 0.049\,\mathrm{mM}$$
$$k_m^{pp} = 0.166\,\mathrm{mM}$$

Pyrophosphate phosphohydrolase v_7 (PPASE)

$$pp + h2o \rightarrow 2\ p$$

$$v_7([pp]) = v_{max} \frac{[pp]}{[pp] + k_m^{pp}} \tag{10}$$

$$\triangle G^{0'} = -19.1\,\mathrm{kJ/mol}$$
$$k_m^{pp} = 0.005\,\mathrm{mM}$$

Glycogen synthase v_8 (GS)

$$udpglc \rightarrow udp + glyglc$$

$$\begin{aligned}
f_{max}([glyglc]) &= (1 + k_1^{max})\,\frac{(C - [glyglc])}{(C - [glyglc]) + k_1^{max} C} \\
v_8^{dp}([udpglc],[glc6p],[glyglc]) &= v_{max} f_{max} \frac{[udpglc]}{[udpglc] + (\frac{k_1^{dp}}{k_2^{dp} + [glc6p]})} \\
v_8^{p}([udpglc],[glc6p],[glyglc]) &= v_{max} f_{max} \frac{[udpglc]}{[udpglc] + (\frac{k_1^{p}}{k_2^{p} + [glc6p]})} \\
v_8([udpglc],[glc6p],[glyglc]) &= (1 - \gamma_{GS}) \cdot v_8^{dp} + \gamma_{GS} \cdot v_8^{p}
\end{aligned} \tag{11}$$

$$\begin{aligned}
k_1^{dp} &= 0.224\,\mathrm{mM}^2 \\
k_2^{dp} &= 0.1504\,\mathrm{mM} \\
k_1^{p} &= 3.003\,\mathrm{mM}^2 \\
k_2^{p} &= 0.09029\,\mathrm{mM} \\
k_1^{max} &= 0.2 \\
C &= 500\,\mathrm{mM}
\end{aligned}$$

Glycogen phosphorylase v_9 (GP)

$$glyglc + p \leftrightarrow glc1p$$

$$\begin{aligned}
f_{max}([glyglc]) &= (1 + k_1^{max})\,\frac{[glyglc]}{[glyglc] + k_1^{max} C} \\
v_{max}^{dp}([amp]) &= v_{max} f_{max} \left(k_{dp,base}^{amp} + (k_{dp,max}^{amp} - k_{dp,base}^{amp}) \left(\frac{[amp]}{[amp] + k_{dp,a}^{amp}} \right) \right) \\
v_{max}^{p}([glc]) &= v_{max} f_{max} \exp(-\frac{\ln(2)}{k_{p,i}^{glc}} [glc])
\end{aligned}$$

$$\begin{aligned}
v_9^{dp}([glyglc],[p],[glc1p],[amp]) &= \frac{\frac{v_{max}^{dp}}{k_{dp}^{glyc} k_{dp}^{p}} \left([glyglc][P] - \frac{[glc1p]}{k_{eq}} \right)}{\left(1 + \frac{[glyglc]}{k_{dp}^{glyc}}\right) \left(1 + \frac{[p]}{k_{dp}^{p}}\right) + \left(\frac{[glc1p]}{k_{dp}^{glc1p}}\right) - 1} \\
v_9^{p}([glyglc],[p],[glc1p],[glc]) &= \frac{\frac{v_{max}^{p}}{k_{p}^{glyc} k_{p}^{p}} \left([glyglc][p] - \frac{[glc1p]}{k_{eq}} \right)}{\left(1 + \frac{[glyglc]}{k_{p}^{glyc}}\right) \left(1 + \frac{[P]}{k_{p}^{p}}\right) + \left(\frac{[glc1p]}{k_{p}^{glc1p}}\right) - 1} \\
v_9([glyglc],[p],[glc1p],[amp],[glc]) &= (1 - \gamma_{GP}) \cdot v_9^{dp} + \gamma_{GP} \cdot v_9^{p}
\end{aligned} \tag{12}$$

$$
\begin{aligned}
\triangle G^{0'} &= 4.0\,\mathrm{kJ/mol} \\
k_{eq} &= 0.21\,\frac{1}{\mathrm{mM}} \\
k_{dp}^{glyc} &= 4.8\,\mathrm{mM} \\
k_{p}^{glyc} &= 2.7\,\mathrm{mM} \\
k_{dp}^{glc1p} &= 120\,\mathrm{mM} \\
k_{p}^{glc1p} &= 2\,\mathrm{mM} \\
k_{dp}^{p} &= 300\,\mathrm{mM} \\
k_{p}^{p} &= 5\,\mathrm{mM} \\
k_{p,i}^{glc} &= 5\,\mathrm{mM} \\
k_{dp,a}^{amp} &= 1\,\mathrm{mM} \\
k_{dp,base}^{amp} &= 0.03 \\
k_{dp,max}^{amp} &= 0.30 \\
k_{1}^{max} &= 0.2 \\
C &= 500\,\mathrm{mM}
\end{aligned}
$$

Nucleosid diphosphate kinase (GTP, UTP) $v_{10,11}$ (NDK)

$$atp + gdp \leftrightarrow adp + gtp$$

$$atp + udp \leftrightarrow adp + utp$$

$$v_{10}([atp],[adp],[gtp],[gdp]) = \frac{\frac{v_{max}}{k_m^{atp} k_m^{gdp}}\left([atp][gdp] - \frac{[adp][gtp]}{k_{eq}}\right)}{(1+\frac{[atp]}{k_m^{atp}})(1+\frac{[gdp]}{k_m^{gdp}}) + (1+\frac{[adp]}{k_m^{adp}})(1+\frac{[gtp]}{k_m^{gtp}}) - 1} \tag{13}$$

$$v_{11}([atp],[adp],[utp],[udp]) = \frac{\frac{v_{max}}{k_m^{atp} k_m^{udp}}\left([atp][udp] - \frac{[adp][utp]}{k_{eq}}\right)}{(1+\frac{[atp]}{k_m^{atp}})(1+\frac{[udp]}{k_m^{udp}}) + (1+\frac{[adp]}{k_m^{adp}})(1+\frac{[utp]}{k_m^{utp}}) - 1} \tag{14}$$

$$
\begin{aligned}
\triangle G^{0'} &= 0\,\mathrm{kJ/mol} \\
k_{eq} &= 1 \\
k_m^{atp} &= 1.33\,\mathrm{mM} \\
k_m^{adp} &= 0.042\,\mathrm{mM} \\
k_m^{utp} &= 16\,\mathrm{mM} \\
k_m^{udp} &= 0.19\,\mathrm{mM} \\
k_m^{gtp} &= 0.15\,\mathrm{mM} \\
k_m^{gdp} &= 0.031\,\mathrm{mM}
\end{aligned}
$$

Adenylate kinase v_{12} (AK)

$$atp + amp \leftrightarrow 2\ adp$$

$$v_{12}([atp],[adp],[amp]) = \frac{\frac{v_{max}}{k_m^{atp} k_m^{amp}}\left([atp][amp] - \frac{[adp]^2}{k_{eq}}\right)}{\left(1+\frac{[atp]}{k_m^{atp}}\right)\left(1+\frac{[amp]}{k_m^{amp}}\right) + \frac{2[adp]}{k_m^{adp}} + \frac{[adp]^2}{(k_m^{adp})^2}} \tag{15}$$

$$\triangle G^{0'} = 3.6\,\mathrm{kJ/mol}$$
$$k_{eq} = 0.25$$
$$k_m^{atp} = 0.09\,\mathrm{mM}$$
$$k_m^{amp} = 0.08\,\mathrm{mM}$$
$$k_m^{adp} = 0.11\,\mathrm{mM}$$

Phosphofructo kinase 2 v_{13} (PFK2)

$$atp + fru6p \rightarrow adp + fru26bp$$

$$\begin{aligned}
v_{13}^{dp}([fru6p],[atp]) &= v_{max}^{dp} \cdot \frac{[fru6p]^{n_{dp}}}{[fru6p]^{n_{dp}} + (k_{dp}^{fru6p})^{n_{dp}}} \cdot \frac{[atp]}{[atp] + k_{dp}^{atp}} \\
v_{13}^{p}([fru6p],[atp]) &= v_{max}^{p} \cdot \frac{[fru6p]^{n_p}}{[fru6p]^{n_p} + (k_p^{fru6p})^{n_p}} \cdot \frac{[atp]}{[atp] + k_p^{atp}} \\
v_{13}([fru6p],[atp]) &= (1 - \gamma_{pfk2}) \cdot v_{13}^{dp} + \gamma_{pfk2} \cdot v_{13}^{p}
\end{aligned} \tag{16}$$

$$\triangle G^{0'} = -14.2\,\mathrm{kJ/mol}$$
$$n_{dp} = 1.3$$
$$n_p = 2.1$$
$$k_{dp}^{fru6p} = 0.016\,\mathrm{mM}$$
$$k_p^{fru6p} = 0.050\,\mathrm{mM}$$
$$k_{dp}^{atp} = 0.28\,\mathrm{mM}$$
$$k_p^{atp} = 0.65\,\mathrm{mM}$$
$$v_{max}^{p} = v_{max}^{n}$$

Fructose-2,6-bisphosphatase v_{14} (FBP2)

$$fru26bp + h2o \rightarrow p + fru6p$$

$$\begin{aligned}
v_{14}^{dp}([fru6p],[fru26bp]) &= \frac{v_{max}^{dp}}{1 + \frac{[fru6p]}{k_{i,dp}^{fru6p}}} \cdot \frac{[fru26bp]}{k_{m,dp}^{fru26bp} + [fru26bp]} \\
v_{14}^{p}([fru6p],[fru26bp]) &= \frac{v_{max}^{p}}{1 + \frac{[fru6p]}{k_{i,p}^{fru6p}}} \cdot \frac{[fru26bp]}{k_{m,p}^{fru26bp} + [fru26bp]} \\
v_{14}^{p} &= (1 - \gamma_{FBP2}) \cdot v_{14}^{dp} + \gamma_{FBP2} \cdot v_{14}^{p}
\end{aligned} \tag{17}$$

$$\triangle G^{0'} = -16.3\,\mathrm{kJ/mol}$$
$$k_{m,dp}^{fru26bp} = 0.010\,\mathrm{mM}$$
$$k_{m,p}^{fru26bp} = 0.0005\,\mathrm{mM}$$
$$k_{i,dp}^{fru6p} = 0.0035\,\mathrm{mM}$$
$$k_{i,p}^{fru6p} = 0.010\,\mathrm{mM}$$
$$\gamma_{FBP2} = \gamma_{PFK2}$$
$$v_{max}^{p} = v_{max}^{dp}$$

Phosphofructo kinase 1 v_{15} (PFK1)

$$atp + fru6p \rightarrow fru16p + adp$$

$$v_{15}([fru6p],[atp],[fru26bp]) = v_{max}\left(1 + \frac{[fru26bp]}{k_a^{fru26bp}}\right) \left(\frac{[fru6p][atp]}{k_i^{fru6p}\cdot k_m^{atp} + k_m^{fru6p}\cdot [atp] + k_m^{atp}\cdot [fru6p] + [atp]\cdot [fru6p]}\right) \tag{18}$$

$$\triangle G^{0'} = -14.2\,\mathrm{kJ/mol}$$
$$k_m^{atp} = 0.111\,\mathrm{mM}$$
$$k_m^{fru6p} = 0.077\,\mathrm{mM}$$
$$k_i^{fru6p} = 0.012\,\mathrm{mM}$$
$$k_a^{fru26bp} = 0.001\,\mathrm{mM}$$

Fructose-1,6-bisphosphatase v_{16} (FBP1)

$$fru16bp + h2o \rightarrow fru6p + p$$

$$v_{16}([fru16bp],[fru26bp]) = \frac{v_{max}}{1 + \frac{[fru26bp]}{k_i^{fru26bp}}}\left(\frac{[fru16bp]}{[fru16bp] + k_m^{fru16bp}}\right) \tag{19}$$

$$\triangle G^{0'} = -16.3\,\mathrm{kJ/mol}$$
$$k_i^{fru26bp} = 0.001\,\mathrm{mM}$$
$$k_m^{fru16bp} = 0.0013\,\mathrm{mM}$$

Aldolase v_{17} (ALD)

$$fru16bp \leftrightarrow dhap + grap$$

$$v_{17}([fru16p],[dhap],[grap]) = \frac{\frac{v_{max}}{k_m^{fru16bp}}\cdot\left([fru16bp] - \frac{[grap][dhap]}{k_{eq}}\right)}{1 + \frac{[fru16bp]}{k_m^{fru16bp}} + \frac{[grap]}{k_{i1}^{grap}} + \frac{[dhap]([grap]+k_m^{grap})}{k_m^{dhap}\cdot k_{i1}^{grap}} + \frac{[fru16bp][grap]}{k_m^{fru16bp}k_{i2}^{grap}}} \tag{20}$$

$$\triangle G^{0'} = 23.8\,\mathrm{kJ/mol}$$
$$k_{eq} = 9.76^{-5}\,\mathrm{mM}$$
$$k_m^{fru16bp} = 0.0071\,\mathrm{mM}$$
$$k_m^{dhap} = 0.0364\,\mathrm{mM}$$
$$k_m^{grap} = 0.0071\,\mathrm{mM}$$
$$k_{i1}^{grap} = 0.0572\,\mathrm{mM}$$
$$k_{i2}^{grap} = 0.176\,\mathrm{mM}$$

Triosephosphate isomerase v_{18} (TPI)

$$dhap \leftrightarrow grap$$

$$v_{18}([dhap],[grap]) = \frac{v_{max}}{k_m^{dhap}} \cdot \left(\frac{[dhap] - \frac{[grap]}{k_{eq}}}{1 + \frac{[dhap]}{k_m^{dhap}} + \frac{[grap]}{k_m^{grap}}} \right) \tag{21}$$

$$\triangle G^{0'} = 7.5\,\mathrm{kJ/mol}$$
$$k_{eq} = 0.0545\,\mathrm{mM}$$
$$k_m^{dhap} = 0.59\,\mathrm{mM}$$
$$k_m^{grap} = 0.42\,\mathrm{mM}$$

D-Glyceraldehyde-3-phosphate:NAD+ oxidoreductase v_{19} (GAPDH, Glyceraldehydephosphate dehydrogenase)

$$grap + p + nad \leftrightarrow nadh + h + bpg13$$

$$v_{19}([grap],[p],[nad],[nadh],[bpg13]) = \frac{\frac{v_{max}}{k_{nad}\cdot k_{grap}\cdot k_p}\left([nad][grap][p] - \frac{[bpg13][nadh]}{k_{eq}}\right)}{\left(1+\frac{[nad]}{k^{nad}}\right)\left(1+\frac{[grap]}{k^{grap}}\right)\left(1+\frac{[p]}{k_p}\right) + \left(1+\frac{[nadh]}{k_{nadh}}\right)\left(1+\frac{[bpg13]}{k_{bpg13}}\right) - 1} \tag{22}$$

$$\triangle G^{0'} = 6.3\,\mathrm{kJ/mol}$$
$$k_{eq} = 0.0868\,\frac{1}{\mathrm{mM}}$$
$$k^{nad} = 0.05\,\mathrm{mM}$$
$$k^{grap} = 0.005\,\mathrm{mM}$$
$$k^{p} = 3.9\,\mathrm{mM}$$
$$k^{nadh} = 0.0083\,\mathrm{mM}$$
$$k^{bpg13} = 0.0035\,\mathrm{mM}$$

Phosphoglycerate kinase v_{20} (PGK)

$$adp + bpg13 \leftrightarrow atp + pg3$$

$$v_{20}([adp],[atp],[bpg13],[pg3]) = \frac{\frac{v_{max}}{k_{adp}k_{bpg13}}\left([adp][bpg13] - \frac{[atp][pg3]}{k_{eq}}\right)}{\left(1+\frac{[adp]}{k_{adp}}\right)\left(1+\frac{[bpg13]}{k_{bpg13}}\right) + \left(1+\frac{[atp]}{k_{atp}}\right)\left(1+\frac{[pg3]}{k_{pg3}}\right) - 1} \tag{23}$$

$$\triangle G^{0'} = -5\,\mathrm{kJ/mol}\ (-18.5\,\mathrm{kJ/mol})$$
$$k_{eq} = 7\ (1310)$$
$$k^{adp} = 0.35\,\mathrm{mM}$$
$$k^{bpg13} = 0.002\,\mathrm{mM}$$
$$k^{atp} = 0.48\,\mathrm{mM}$$
$$k^{pg3} = 1.2\,\mathrm{mM}$$

3-Phosphoglycerate mutase v_{21} (PGM)

$$pg3 \leftrightarrow pg2$$

$$v_{21}([pg3],[pg2]) = \frac{v_{max}\left([pg3] - \frac{[pg2]}{k_{eq}}\right)}{[pg3] + k_{pg3}\left(1 + \frac{[pg2]}{k_{pg2}}\right)} \tag{24}$$

$$\triangle G^{0'} = 4.4\,\mathrm{kJ/mol}$$
$$k_{eq} = 0.1814$$
$$k^{pg3} = 5\,\mathrm{mM}$$
$$k^{pg2} = 1\,\mathrm{mM}$$

Enolase v_{22} (EN)

$$pg2 \leftrightarrow pep + h2o$$

$$v_{22}([pg2],[pep]) = \frac{v_{max}\left([pg2] - \frac{[pep]}{k_{eq}}\right)}{[pg2] + k_{pg2}\left(1 + \frac{[pep]}{k_{pep}}\right)} \tag{25}$$

$$\triangle G^{0'} = 7.5\,\mathrm{kJ/mol}$$
$$k_{eq} = 0.0545$$
$$k^{pg2} = 1\,\mathrm{mM}$$
$$k^{pep} = 1\,\mathrm{mM}$$

Pyruvate kinase v_{23} (PK)

$$adp + pep \rightarrow atp + pyr$$

$$\begin{aligned}
f^{dp} &= \frac{[fru16bp]^{n_{fbp}}}{[fru16bp]^{n_{fbp}} + (k^{dp}_{fbp})^{n_{fbp}}} \\
f^{p} &= \frac{[fru16bp]^{n_{fbp}}}{[fru16bp]^{n_{fbp}} + (k^{dp}_{fbp})^{n_{fbp}}} \\
\alpha^{dp}_{inp} &= (1-f)(\alpha^{dp} - \alpha_{end}) + \alpha_{end} \\
\alpha^{p}_{inp} &= (1-f)(\alpha^{p} - \alpha_{end}) + \alpha_{end} \\
k^{dp}_{pep,inp} &= (1-f)(k^{dp}_{pep} - k^{min}_{pep}) + k^{min}_{pep} \\
k^{p}_{pep,inp} &= (1-f)(k^{p}_{pep} - k^{min}_{pep}) + k^{min}_{pep}
\end{aligned}$$

$$\begin{aligned}
v^{dp}_{23} &= v_{max}\alpha^{dp}_{inp}\left(\frac{[pep]^{n^{pep}}}{[pep]^{n^{pep}} + (k^{dp}_{pep,inp})^{n^{pep}}}\right)\left(\frac{[adp]}{[adp] + k_{adp}}\right)\left(a^{dp}_{base} + (1 - a^{dp}_{base})f^{dp}\right) \\
v^{p}_{23} &= v_{max}\alpha^{p}_{inp}\left(\frac{[pep]^{n^{pep}}}{[pep]^{n^{pep}} + (k^{p}_{pep,inp})^{n^{pep}}}\right)\left(\frac{[adp]}{[adp] + k_{adp}}\right)(a^{p}_{base} + (1 - a^{p}_{base})f^{p}) \\
v_{23}([pep],[adp]) &= (1 - \gamma_{PK}) \cdot v^{dp}_{23} + \gamma_{PK} \cdot v^{p}_{23}
\end{aligned} \tag{26}$$

$$
\begin{aligned}
\triangle G^{0'} &= -31.4\,\mathrm{kJ/mol} \\
n^{pep} &= 3.5 \\
n^{fbp} &= 1.8 \\
k_{pep}^{dp} &= 0.58\,\mathrm{mM} \\
k_{pep}^{p} &= 1.10\,\mathrm{mM} \\
k_{pep}^{min} &= 0.08\,\mathrm{mM} \\
\alpha^{dp} &= 1.0 \\
\alpha^{p} &= 1.1 \\
\alpha_{end} &= 1.0 \\
k_{fbp}^{dp} &= 0.16 \cdot 10^{-3}\,\mathrm{mM} \\
k_{fbp}^{p} &= 0.35 \cdot 10^{-3}\,\mathrm{mM} \\
a_{base}^{dp} &= 0.08 \\
a_{base}^{p} &= 0.04 \\
k_{adp} &= 2.3\,\mathrm{mM}
\end{aligned}
$$

Phosphoenolpyruvate carboxykinase $v_{24,25}$ (PEPCK) Reactions with very similar kinetics in mitochondrion and cytosol.

$$oaa + gtp \leftrightarrow pep + gdp + co2$$

$$v_{24}([oaa],[gtp],[pep],[gdp],[co2]) = \frac{\frac{v_{max}}{k_m^{gtp} k_m^{oaa}} \left([oaa][gtp] - \frac{[pep][gdp][co2]}{k_{eq}}\right)}{\left(1+\frac{[oaa]}{k_m^{oaa}}\right)\left(1+\frac{[gtp]}{k_m^{gtp}}\right) + \left(1+\frac{[pep]}{k_m^{pep}}\right)\left(1+\frac{[gdp]}{k_m^{gdp}}\right)\left(1+\frac{[co2]}{k_m^{co2}}\right) - 1} \quad (27)$$

$$v_{25}([oaa\_mito],[gtp\_mito],[pep\_mito],[gdp\_mito],[co2\_mito]) = \frac{\frac{v_{max}}{k_m^{gtp} k_m^{oaa}} \left([oaa\_mito][gtp\_mito] - \frac{[pep\_mito][gdp\_mito][co2\_mito]}{k_{eq}}\right)}{\left(1+\frac{[oaa\_mito]}{k_m^{oaa}}\right)\left(1+\frac{[gtp\_mito]}{k_m^{gtp}}\right) + \left(1+\frac{[pep\_mito]}{k_m^{pep}}\right)\left(1+\frac{[gdp\_mito]}{k_m^{gdp}}\right)\left(1+\frac{[co2\_mito]}{k_m^{co2}}\right) - 1} \quad (28)$$

$$
\begin{aligned}
\triangle G^{0'} &= -15\,\mathrm{kJ/mol} \\
k_{eq} &= 337 \frac{1}{\mathrm{mM}} \\
k_m^{pep} &= 0.237\,\mathrm{mM} \\
k_m^{gdp} &= 0.0921\,\mathrm{mM} \\
k_m^{co2} &= 25.5\,\mathrm{mM} \\
k_m^{oaa} &= 0.0055\,\mathrm{mM} \\
k_m^{gtp} &= 0.0222\,\mathrm{mM}
\end{aligned}
$$

Pyruvate carboxylase v_{26} (PC)

$$pyr + atp + co2 \rightarrow oaa + adp + p$$

$$v_{26}([pyr\_mito][atp\_mito][co2\_mito][acoa\_mito]) = v_{max}\left(\frac{[pyr\_mito]}{k_m^{pyr} + [pyr\_mito]}\right)\left(\frac{[atp\_mito]}{k_m^{atp} + [atp\_mito]}\right)\left(\frac{[co2\_mito]}{k_m^{co2} + [co2\_mito]}\right)\left(\frac{[acoa\_mito]^n}{(k_m^{acoa})^n + [acoa\_mito]^n}\right) \tag{29}$$

$$k_m^{atp} = 0.22\,\text{mM}$$
$$k_m^{pyr} = 0.22\,\text{mM}$$
$$k_m^{co2} = 3.2\,\text{mM}$$
$$k^{acoa} = 0.015\,\text{mM}$$
$$n = 2.5$$

Lactate dehydrogenase v_{27} (LDH)

$$pyr + nadh + h \leftrightarrow lac + nad$$

$$v_{27}([lac],[pyr],[nadh],[nad]) = \frac{\frac{v_{max}}{k_m^{pyr} k_m^{nadh}}\left([pyr][nadh] - \frac{[lac][nad]}{k_{eq}}\right)}{\left(1 + \frac{[nadh]}{k_m^{nadh}}\right)\left(1 + \frac{[pyr]}{k_m^{pyr}}\right) + \left(1 + \frac{[lac]}{k_m^{lac}}\right)\left(1 + \frac{[nad]}{k_m^{nad}}\right) - 1} \tag{30}$$

$$\triangle G^{0'} = 21.1\,\text{kJ/mol}$$
$$k_{eq} = 2.78 \cdot 10^{-4}$$
$$k_m^{pyr} = 0.495\,\text{mM}$$
$$k_m^{lac} = 31.98\,\text{mM}$$
$$k_m^{nad} = 0.984\,\text{mM}$$
$$k_m^{nadh} = 0.027\,\text{mM}$$

Lactate transporter v_{28} (LACT)

$$lac\_ext \leftrightarrow lac$$

$$v_{28}([lac],[lac\_ext]) = \frac{\frac{v_{max}}{k_m^{lac}}\left([lac\_ext] - \frac{[lac]}{k_{eq}}\right)}{1 + \frac{[lac\_ext]}{k_m^{lac}} + \frac{[lac]}{k_m^{lac}}} \tag{31}$$

$$\triangle G^{0'} = 0\,\text{kJ/mol}$$
$$k_{eq} = 1$$
$$k_m^{lac} = 0.8\,\text{mM}$$

Pyruvate transporter v_{29} (PYRT)

$$pyr \leftrightarrow pyr\_mito$$

$$v_{29}([pyr],[pyr\_mito]) = \frac{\frac{v_{max}}{k_m^{pyr}}\left([pyr] - \frac{[pyr\_mito]}{k_{eq}}\right)}{1 + \frac{[pyr\_mito]}{k_m^{pyr}} + \frac{[pyr]}{k_m^{pyr}}} \tag{32}$$

$$\triangle G^{0'} = 0\,\mathrm{kJ/mol}$$
$$k_{eq} = 1$$
$$k_m^{pyr} = 0.1\,\mathrm{mM}$$

PEP transporter v_{30} (PEPT)

$$pep\_mito \leftrightarrow pep$$

$$v_{30}([pep\_mito],[pep]) = \frac{\frac{v_{max}}{k_m^{pep}}\left([pep\_mito] - \frac{[pep]}{k_{eq}}\right)}{1 + \frac{[pep\_mito]}{k_m^{pep}} + \frac{[pep]}{k_m^{pep}}} \quad (33)$$

$$\triangle G^{0'} = 0\,\mathrm{kJ/mol}$$
$$k_{eq} = 1$$
$$k_m^{pep} = 0.1\,\mathrm{mM}$$

Pyruvate dehydrogenase v_{31} (PDH)

$$pyr\_mito + coa\_mito + nad \rightarrow acoa + co2 + nadh + h$$

$$v_{31}^{dp}([pyr],[coa],[nad],[nadh],[acoa]) =$$
$$v_{max}\alpha_{dp}\left(\frac{[pyr]}{[pyr]+k^{pyr}}\right)\left(\frac{[nad]}{[nad]+k^{nad}(1+\frac{[nadh]}{k_i^{nadh}})}\right)\left(\frac{[coa]}{[coa]+k^{coa}(1+\frac{[acoa]}{k_i^{acoa}})}\right)$$

$$v_{31}^{p}([pyr],[coa],[nad],[nadh],[acoa]) =$$
$$v_{max}\alpha_{p}\left(\frac{[pyr]}{[pyr]+k^{pyr}}\right)\left(\frac{[nad]}{[nad]+k^{nad}(1+\frac{[nadh]}{k_i^{nadh}})}\right)\left(\frac{[coa]}{[coa]+k^{coa}(1+\frac{[acoa]}{k_i^{acoa}})}\right)$$

$$v_{23}([pyr],[coa],[nadh],[nad],[acoa]) = (1-\gamma_{PDH})\cdot v_{31}^{dp} + \gamma_{PDH}\cdot v_{31}^{p} \quad (34)$$

$$k^{pyr} = 0.025\,\mathrm{mM}$$
$$k^{coa} = 0.013\,\mathrm{mM}$$
$$k^{nad} = 0.050\,\mathrm{mM}$$
$$k_i^{acoa} = 0.035\,\mathrm{mM}$$
$$k_i^{nadh} = 0.036\,\mathrm{mM}$$
$$\alpha_{dp} = 5\,\mathrm{mM}$$
$$\alpha_{p} = 1\,\mathrm{mM}$$

Citrate synthase v_{32} (CS)

$$oaa\_mito + acoa\_mito + h2o\_mito \leftrightarrow cit\_mito + coa\_mito$$

$$v_{32}([oaa\_mito],[acoa\_mito],[cit\_mito],[coa\_mito]) =$$
$$\frac{\frac{v_{max}}{k^{oaa}k^{acoa}}\left([oaa\_mito][acoa\_mito] - \frac{[cit\_mito][coa\_mito]}{k_{eq}}\right)}{\left(1+\frac{[oaa\_mito]}{k^{oaa}}\right)\left(1+\frac{[acoa\_mito]}{k^{acoa}}\right)+\left(1+\frac{[cit\_mito]}{k^{cit}}\right)\left(1+\frac{[coa\_mito]}{k^{coa}}\right)-1} \quad (35)$$

$$\triangle G^{0'} = -32.2\,\mathrm{kJ/mol}$$
$$k_{eq} = 2.66E5$$
$$k^{oaa} = 0.002\,\mathrm{mM}$$
$$k^{acoa} = 0.016\,\mathrm{mM}$$
$$k^{cit} = 0.420\,\mathrm{mM}$$
$$k^{coa} = 0.070\,\mathrm{mM}$$

Nucleosid diphosphate kinase v_{33} (GTP, UTP) (NDK)

$$atp\_mito + gdp\_mito \leftrightarrow adp\_mito + gtp\_mito$$

$$v_{33}([atp\_mito],[adp\_mito],[gtp\_mito],[gdp\_mito]) = \frac{\frac{v_{max}}{k_m^{atp}k_m^{gdp}}\left([atp\_mito][gdp\_mito] - \frac{[adp\_mito][gtp\_mito]}{k_{eq}}\right)}{(1+\frac{[atp]}{k_m^{atp}})(1+\frac{[gdp\_mito]}{k_m^{gdp}}) + (1+\frac{[adp\_mito]}{k_m^{adp}})(1+\frac{[gtp\_mito]}{k_m^{gtp}}) - 1} \tag{36}$$

$$\triangle G^{0'} = 0\,\mathrm{kJ/mol}$$
$$k_{eq} = 1$$
$$k_m^{atp} = 1.33\,\mathrm{mM}$$
$$k_m^{adp} = 0.042\,\mathrm{mM}$$
$$k_m^{gtp} = 0.15\,\mathrm{mM}$$
$$k_m^{gdp} = 0.031\,\mathrm{mM}$$

Oxalacetate flux v_{34} (OAAFLX)

$$\rightarrow oaa\_mito$$

$$v_{34} = v_{max} \tag{37}$$

Acetyl-CoA flux v_{35} (ACOAFLX)

$$acoa\_mito \rightarrow$$

$$v_{35} = v_{max} \tag{38}$$

Citrate flux v_{36} (CITFLX)

$$cit\_mito \rightarrow$$

$$v_{36} = v_{max} \tag{39}$$
