# Supplementary material for: Quantifying the Contribution of the Liver to Glucose Homeostasis: A Detailed Kinetic Model of Human Hepatic Glucose Metabolism
Source: PLoS Comput Biol. 2012 Jun 21;8(6):e1002577. doi: 10.1371/journal.pcbi.1002577 (PMC3383054; doi:10.1371/journal.pcbi.1002577)
Supplement: Text S4 — Overview allosteric regulations. The respective rate equations are given (Text S3). Allosterically regulated enzymes characteristic for either HGP or HGU are marked respectively. Allosteric regulators can function as activators are inhibitors. The references stating the roles of the allosteric effectors and the parameters in the rate equations are given. (PDF) [file pcbi.1002577.s015.pdf]

Text S4. Overview allosteric regulations.

Enzyme	Equation	HGP/HGU	Allosteric Effector	Effect	References
GK	eq.5	HGU	glc	activation	[1,2]
GK	eq.5	HGU	fru6p	inhibition	[1,2]
PK	eq.26	HGU	fru16bp	activation	[3,4]
PFK1	eq.18	HGU	fru26bp	activation	[5-7]
FBP1	eq.19	HGP	fru26bp	inhibition	[5,6,8]
FBP2	eq.17	HGP	fru6p	inhibition	[9,10]
PC	eq.29	HGP	acoa	activation	[11,12]
PDH	eq.34		acoa	inhibition	[2,13]
GP [dp]	eq.12	HGP	amp	activation	[14,15]
GP [p]	eq.12	HGP	glc	inhibition	[1,15]
GS	eq.11	HGU	glc6p	activation	[1,16]

References

1. Agius L (2008) Glucokinase and molecular aspects of liver glycogen metabolism. Biochem J 414: 1-18.
2. Nelson DL, Cox MM, Lehninger AL (2008) Principles of biochemistry. New York: Freeman. getr. Zählung. p.
3. Flory W, Peczon BD, Koeppe RE, Spivey HO (1974) Kinetic properties of rat liver pyruvate kinase at cellular concentrations of enzyme, substrates and modifiers. Biochem J 141: 127-131.
4. Ishibashi H, Cottam GL (1978) Glucagon-stimulated phosphorylation of pyruvate kinase in hepatocytes. J Biol Chem 253: 8767-8771.
5. Pilkis SJ, Granner DK (1992) Molecular physiology of the regulation of hepatic gluconeogenesis and glycolysis. Annu Rev Physiol 54: 885-909.
6. Rider MH, Bertrand L, Vertommen D, Michels PA, Rousseau GG, et al. (2004) 6-phosphofructo-2-kinase/fructose-2,6-bisphosphatase: head-to-head with a bifunctional enzyme that controls glycolysis. Biochem J 381: 561-579.
7. Van Schaftingen E, Jett MF, Hue L, Hers HG (1981) Control of liver 6-phosphofructokinase by fructose 2,6-bisphosphate and other effectors. Proc Natl Acad Sci U S A 78: 3483-3486.
8. Adams A, Redden C, Menahem S (1990) Characterization of human fructose-1,6-bisphosphatase in control and deficient tissues. J Inherit Metab Dis 13: 829-848.
9. Lee YH, Li Y, Uyeda K, Hasemann CA (2003) Tissue-specific structure/function differentiation of the liver isoform of 6-phosphofructo-2-kinase/fructose-2,6-bisphosphatase. J Biol Chem 278: 523-530.
10. Sakakibara R, Kitajima S, Uyeda K (1984) Differences in kinetic properties of phospho and dephospho forms of fructose-6-phosphate, 2-kinase and fructose 2,6-bisphosphatase. J Biol Chem 259: 41-46.
11. Jitrapakdee S, St Maurice M, Rayment I, Cleland WW, Wallace JC, et al. (2008) Structure, mechanism and regulation of pyruvate carboxylase. Biochem J 413: 369-387.
12. Jitrapakdee S, Walker ME, Wallace JC (1999) Functional expression, purification, and characterization of recombinant human pyruvate carboxylase. Biochem Biophys Res Commun 266: 512-517.
13. Kiselevsky YV, Ostrovtsova SA, Strumilo SA (1990) Kinetic characterization of the pyruvate and oxoglutarate dehydrogenase complexes from human heart. Acta Biochim Pol 37: 135-139.
14. Lederer B, Stalmans W (1976) Human liver glycogen phosphorylase. Kinetic properties and assay in biopsy specimens. Biochem J 159: 689-695.
15. Stalmans W, Gevers G (1981) The catalytic activity of phosphorylase b in the liver. With a note on the assay in the glycogenolytic direction. Biochem J 200: 327-336.
16. Gerich JE (1993) Control of glycaemia. Baillieres Clin Endocrinol Metab 7: 551-586.
