# Supplementary material for: Quantifying the Contribution of the Liver to Glucose Homeostasis: A Detailed Kinetic Model of Human Hepatic Glucose Metabolism
Source: PLoS Comput Biol. 2012 Jun 21;8(6):e1002577. doi: 10.1371/journal.pcbi.1002577 (PMC3383054; doi:10.1371/journal.pcbi.1002577)
Supplement: Text S5 — Overview interconvertible enzymes of human hepatic glucose metabolism. Overview over the alterations in kinetic properties as a consequence of changed phosphorylation state between phosphorylated and dephosphorylated state of key interconvertible enzymes of HGP and HGU. (PDF) [file pcbi.1002577.s016.pdf]

Text S5. Overview interconvertible enzymes of human hepatic glucose metabolism.

Enzyme	HGP /HGU	Dephosphorylated (dp)	Phosphorylated (p)	Phosphorylation Change
**GS**	**HGU**			**Inactivation**
		lower affinity for udpglc	higher affinity for udpglc	lower affinity for substrate
				different activation by glc6p
**GP**	**HGP**			**Activation**
		lower V_{max}	higher V_{max}	increased V_{max}
		no effect glc	inactivation by glc	different activators, inhibitors
		activation by amp	no effect amp	
		little lower affinity glycogen	little higher affinity glycogen	changed affinities substrates
		much lower affinity glc1p	much higher affinity glc1p	and products
		much lower affinity p	much higher affinity p	
**FB2**	**HGP**			**Activation**
		lower affinity for fru26bp	higher affinity for fru26bp	higher affinity for substrate
		higher affinity for inactivator fru6p	lower affinity for inactivator fru6p	lower inactivation by fru6p
**PFK2**	**HGU**			**Inactivation**
		higher affinity for fru6p	lower affinity for fru6p	lower affinity for substrates
		higher affinity for atp	lower affinity for atp	
**PDH**	**HGU**			**Inactivation**
		higher V_{max}	lower V_{max}	decreased V_{max}
**PK**	**HGU**			**Inactivation**
		higher affinity for pep	lower affinity for pep	lower affinity for substrate
		higher affinity for activator fru16p	lower affinity for activator fru16p	lower activation by fru16p
